# Determining the Molecular Shape of Progesterone: Insights from Laser Ablation Rotational Spectroscopy

**DOI:** 10.1021/acs.jpclett.4c03618

**Published:** 2025-02-27

**Authors:** Aran Insausti, Elena R. Alonso, Sofía Municio, Iker León, Lucie Kolesniková, Santiago Mata

**Affiliations:** † Departamento de Química-Física, Facultad de Ciencia y Tecnología, 16402 Universidad del País Vasco (UPV/EHU), 48940 Leioa, Spain; ‡ Instituto Biofisika (UPV/EHU, CSIC), University of the Basque Country, 48940 Leioa, Spain; § Grupo de Espectrocopía Molecular (GEM), Edificio Quifima, Área de Química-Física, Laboratorios de Espectroscopia y Bioespectroscopia, Parque Científico UVa, Unidad Asociada CSIC, Universidad de Valladolid, 47011 Valladolid, Spain; ∥ Department of Analytical Chemistry, University of Chemistry and Technology, Technická 5, 166 28 Prague 6, Czech Republic

## Abstract

Herein, we present the first experimental observation of isolated progesterone, an endogenous steroid, placed in the gas phase by laser ablation and characterized in a supersonic expansion by Fourier transform microwave techniques. Guided by quantum-chemical calculations, we assigned the rotational spectrum of the most stable structure. The internal rotation of the acetyl methyl group led to the observation of A-E doublets in the spectrum, which were analyzed, resulting in a V_3_ barrier of 2.4425 ± 0.0025 kJ mol^–1^. By fitting over 250 transitions, we determined accurate rotational constants that enabled us to compare the gas phase geometrical parameters with those of crystalline forms and complexes with progesterone receptors. Our results indicate that the A ring of progesterone that contains the ketone group is surprisingly flexible, despite its rigid appearance. This finding is particularly significant, since this ring is an active biological site that is involved in strong intermolecular interactions. Notably, progesterone C_21_H_30_O_2_ is the largest molecule investigated using laser ablation rotational spectroscopy.

## Graphical abstract

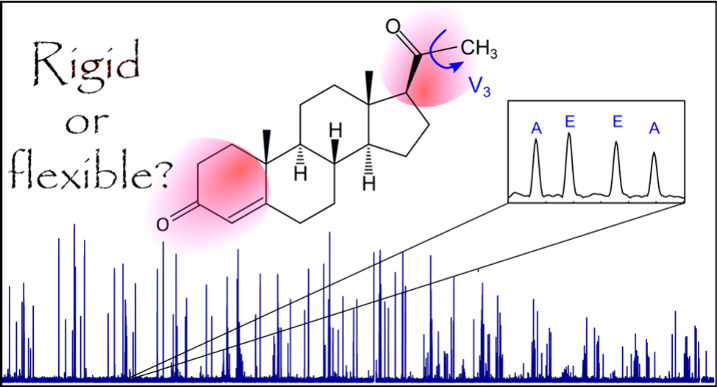

Sex hormones are steroids and encompass many structurally similar compounds, known as progestogens (**Ps**). They interact with progesterone receptors (**PR**) that regulate ovulation and pregnancy in mammalian females.^1,2^ The PRs comprise a superfamily of receptors with distinct biological activities, making their biochemistry a complex subject to study.^3–5^ Apart from binding to progesterone receptors, progestogens exhibit affinity for androgen, glucocorticoid, mineralocorticoid, and even gamma-aminobutyric acid receptors and other non-genomic receptors,^6–10^ resulting in a wide range of biological effects on the brain, bones, metabolism, cardiovascular system, immune system, and others. These substances are commonly used in hormone therapy to manage bothersome menopausal symptoms and urogenital atrophy. However, there is compelling evidence linking the use of certain **Ps** and the irregular expression of **PRs** to the presence of different diseases, such as breast cancer and some autoimmune diseases.^11–14^

Progesterone (**P**) (Scheme 1) is one of the primary and simplest molecules of the **Ps** family. It is an ovarian steroid hormone essential for normal breast development during puberty and in preparation for lactation and breastfeeding. The actions of progesterone are primarily mediated by its high-affinity receptors, which include the classical progesterone receptor (PR)-A and -B isoforms, located in diverse tissues, including the brain, where progesterone controls reproductive behavior, and the breast and reproductive organs.^2^ Several state-of-the-art research methods have been employed to comprehend the biological role of **PRs** and **Ps**. The experimental characterization of the crystal structure of **P** complexed with a progesterone receptor was a significant milestone in understanding these molecules’ biological structure and activity. This information has been crucial for comprehending biological pathways and guiding the development of various pharmacological agents.^15^ Despite numerous studies that have demonstrated the potential for new treatments and therapies based on **P** and its derivatives,^13^ our understanding of the biological activity of **P** remains limited.^16^ The inherent affinity of **Ps** for different receptors and the multitude of parallel processes in which they are involved make these studies genuinely challenging. Typically, biomolecules can adopt multiple conformations, but their structural preferences are often influenced by their environment. Gas-phase investigations provide a distinct advantage by allowing the study of a molecule’s intrinsic structure without external interactions, serving as a fundamental starting point for uncovering their underlying mechanisms. Experiments in jet expansion conditions, for example, play a crucial role in revealing the intrinsic structural behavior by providing information on phenomena that are not easily observable in the condensed environment, such as nondistorted intrinsic structure,^17^ microsolvation,^18–20^ tunneling processes,^21,22^ and tautomeric differentiation.^23,24^

**Scheme 1:**
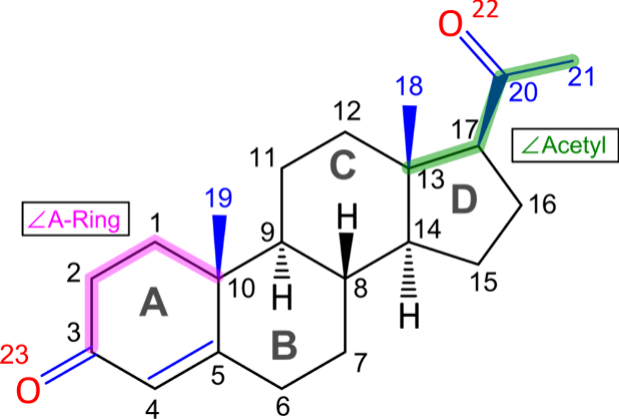
Black Carbon Backbone Represents the General Framework of Steroid Molecules, and the Blue Corresponds to the Specific Parts of the Progesterone Molecule while Magenta and Green Colors Represent the ∠ A-Ring and ∠ Acetyl Dihedral Angles

Advanced rotational spectroscopic techniques in supersonic expansions have implemented short and intense microwave chirps in broadband excitation schemes, as in chirped-pulse Fourier transform microwave (CP-FTMW) spectroscopy, making it possible to record rotational spectra of complex flexible molecules spanning several GHz in a single acquisition.^25^ Rotational spectra enable the unambiguous identification of molecular species, owing to their unique moments of inertia. This technique has recently been used to obtain highly accurate molecular structures of steroid hormones like estrogens (β-estradiol and estrone)^26,27^ and androgen (androsterone)^28^ using the conventional heating vaporization methods. However, we observed that the thermal vaporization processes used in these studies did not bring some steroids to the gas phase. The structural characterization of an essential androgen, testosterone,^29^ was only possible by combining the (CP-FTMW) spectroscopy events with ultrashort laser ablation (LA) pulses in the vaporization processes.

The gas phase structure of neither **Ps** nor progesterone, the primary natural progestogen in vertebrates, was experimentally determined using high-resolution spectroscopy techniques. Therefore, to better understand the molecular affinity of these compounds with their receptors in conformational terms, the objective of this work is, for the first time, to assess experimentally the conformational landscape of progesterone in the gas phase using the above high-resolution spectroscopy. Rotational spectroscopy offers significant advantages but also faces challenges as a general spectroscopic technique. These include the need to volatilize the sample to introduce it into the pulsed molecular beam, the requirement of a nonzero dipole moment, and decreasing sensitivity as molecular size increases due to the rapid increase in the rotational partition function. The cold molecular jet brings the molecules to rotational temperatures below 2 K, which for a molecular system of this size (C_21_H_30_O_2_, 314.46 g·mol^–1^, melting point ∼126 °C), the strongest rotational transitions are situated at the S and C bands (between 2 and 8 GHz). We have overcome these challenges, and here we report the first rotational study of progesterone using a constructed Laser Ablation Fourier transform microwave spectrometer described elsewhere.^30^

The configurational space of **P** should be carefully and efficiently explored first to identify candidates for possible conformers. Our study begins by exploring the theoretical conformational landscape of progesterone using a two-step method combining molecular mechanics and quantum chemistry, which has been demonstrated to be efficient in previous research.^17^ In the first step, all possible molecular configurations were evaluated using the Monte Carlo method based on atomic redistribution and energetic optimization through molecular mechanics, implemented in the Maestro software (Schrödinger 2017).^31^ We also employed our chemical intuition to draw conformers representing stable structures. In the second step, all structures from the first step were optimized using density functional theory (DFT) calculations with the B3LYP-D3BJ^32–34^ method, employing the def2-TZVP^35^ basis sets. The optimized structures were then used to calculate single-point energy using the domain-based local pair natural orbital coupled cluster method with perturbative triple excitations (DLPNO-CCSD­(T))^36^ with the def2-TZVPP basis set. Further details and references of the theoretical calculations can be found in the Supporting Information (SI, Computational Methodology). Using this methodology, we identify six candidates within an energy window of 13 kJ mol^–1^, belonging to two families of conformers exhibiting different configurations of the A ring. These configurations arise from the two possible conformations that the dihedral angle C10–C1–C2–C3 (∠ A-Ring) can adopt (Scheme 1). The first family (I) features a bent configuration for the A-ring of progesterone (Figure 1), with ∠ A-Ring ≈ −55°, while the family (II) presents a twisted form with ∠ A-Ring ≈ 55°. Notably, the rigidity of the rest of the molecule arises from their chair configuration of six-membered rings B and C and the bent disposition in the five-membered ring D with C17 out of the plane, allowing the equatorial arrangement of the acetyl side chain.

**Figure 1:**
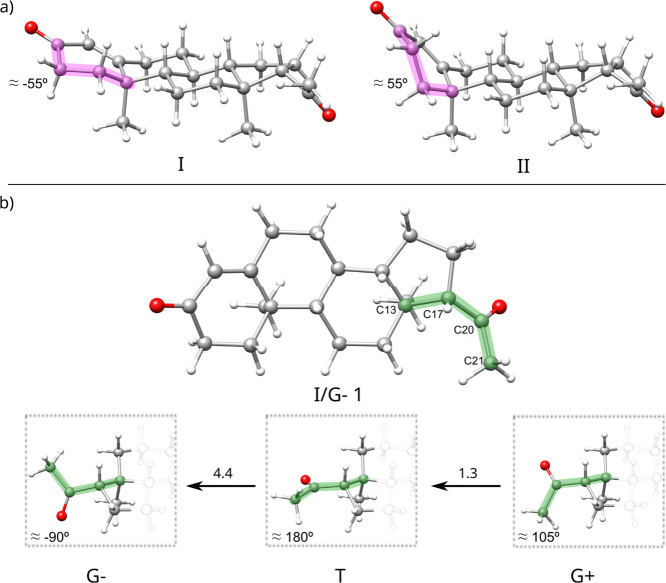
a) The most stable conformers, **I/G- 1** and **II/G- 4**, of the family I and II, respectively. b) Three stable dispositions are adopted by the acetyl group in each family. Arrows and numbers represent the possible interconversion pathway and energy gap in kJ·mol^–1^. The interconversion barrier is virtually the same for both families (all information in SI Figure S1).

Each family presents three stable structures corresponding to the acetyl side chain’s three stable dispositions (the structures’ atomic coordinates can be found in Table S1 of SI). The conformers are labeled according to their family (I or II) and acetyl side chain configuration with the C13–C17–C20–C21 dihedral angle (∠ Acetyl): G– ≈ −90, T ≈ 180, and G+ ≈ 105 degrees (Scheme 1 and Figure 1). Additionally, we introduce another structural parameter considering that the carbon atoms of B, C and D rings maintain the same relative position in all stable structures. Thus, we defined a mean plane based on the carbon atoms of B, C and D rings (λ_BCD_). Furthermore, due to the presence of a carbonyl group (C3=O) in resonance with the C4=C5 double bond, C2, C3, C4, C5, C6, C10, and the O23 atoms in the A ring lie in the same plane. We utilized these atoms to define a second mean plane (λ_A_). The angle between the planes λ_BCD_ and λ_A_ represents the disposition of the A ring concerning the B, C, and D rings, referred to as ϕ_BCD‑A_ in this study. Notably, the conformers of family I exhibit ϕ_BCD‑A_ ≈ 30°, and those of family II exhibit ϕ_BCD‑A_ ≈ 50°. The subsequent results and discussion will use all of the structural parameters reported here.

In the subsequent phase, we proceeded to the experimental part of the research. We employed a custom-built 2–6 GHz CP-FTMW spectrometer featuring a Nd:YAG picosecond laser ablation system (LA-CP-FTMW) operating at 355 nm and 35 ps to capture the rotational spectra of pure progesterone.^30^ Following various experimental optimizations, we acquired and averaged approximately 70,000 molecular free induction decays. It is worth noting that the nonexpanded residual sample resulting from the vaporization of progesterone with laser ablation accumulates at the nozzle outlet, restricting expansion within the chamber to a relatively short period compared to conventional vaporization methods. Consequently, extending the averaging time for the molecular rotational resonant emission signal was not feasible.

The broadband microwave spectrum of **P** is depicted in Figure 2, displayed as the upper trace in black. To identify the spectral signatures of **P**, wide-frequency searches for spectral patterns were carried out based on the rotational constants predicted by theoretical calculations for the six lower-energy conformers (Table 1). Initially, a very intense *a*-type R-branch set of transitions, spanning from J = 6 to 24, was identified and measured, yielding a preliminary set of rotational constants. These constants subsequently enabled the identification of weak *b*-type transitions. Transitions belonging to *c*-type spectra were also searched for but not observed. A total of 121 measured transitions were analyzed using the rigid rotor Hamiltonian as implemented in Pickett’s program,^38^ and the final experimental set of rotational constants (*A, B, C*) and other fit parameters are in Table 1. The best signal-to-noise ratio in the detected lines is ∼120, rendering it impossible to detect rare isotopologues (^13^C and ^18^O) of the observed rotamer, and although considerable efforts were made to detect other conformers of **P**, no new sets of transitions were discovered in the broadband spectrum. However, there were still remnant lines in the spectrum. Concretely, it is worth noting the presence of splitting for certain transitions. The segment in the bottom left panel of Figure 2 illustrates a portion of the rotational spectrum focusing on the J' = 13 and K_a_ = 3 a-type R-branch transitions splitting.

**Figure 2:**
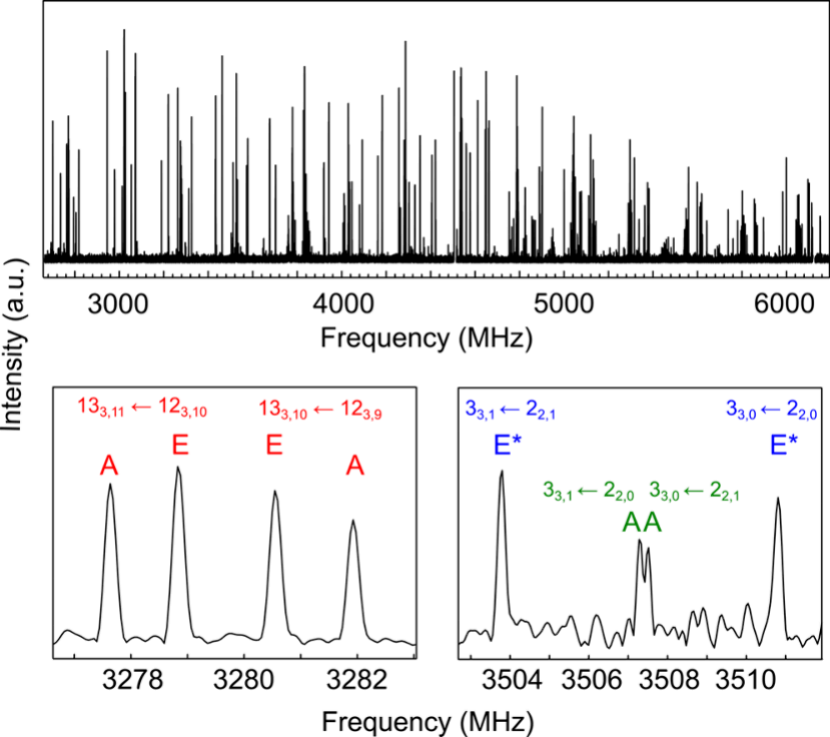
Experimental spectra with A and E splitting caused by the methyl rotor. *a*-type, *b*-type and forbidden *c*-type transitions are in red, green, and blue, respectively.

**Table 1:** Predicted Spectroscopic Parameters for the Six Most Stable Conformers of **P** at the B3LYP-D3BJ/def2-TZVP Level of Theory, along with the Experimental Fitted Values for the Observed Rotamer

	theoretical						experimental
conformer	**I/G- 1**	**I/T 2**	**I/G+ 3**	**II/G- 4**	**II/T 5**	**II/G+ 6**	*A-symmetry state*
*A* ^a^	681	697	689	588	592	596	676.30846 (41)^b^
*B*	131	130	132	141	139	142	131.250945 (71)
*C*	121	119	121	133	132	132	120.446229 (49)
σ (kHz)							10.6
*N* (exp lines)							121
μ_a_|μ_b_|μ_c_	2.4|0.6|0.1	4.5|2.4|0.8	4.0|2.7|2.7	1.9|0.3|1.1	4.0|2.7|1.2	3.8|3.4|2.9	++ | - | No^c^
Δ*E*_CCSD(T)_	0.0	4.4	5.7	5.5	9.7	11.1	
Δ*E*_0_	0.0	4.1	6.2	6.8	10.7	12.9	
Δ*G* ^298K^	0.0	4.4	7.2	7.4	11.6	14.4	
∠ Acetyl	–88.95	168.42	106.46	–88.93	168.44	106.56	
ϕ _BCD‑A_	30.0	29.6	29.6	52.1	53.3	51.8	
∠ A-Ring	–54.70	–54.62	–54.63	55.88	55.90	55.93	

a Rotational constants in the principal axis of inertia in MHz: the theoretical values are from the equilibrium structure, and the experimental values are obtained from the A-symmetry state of *Pickett*’s program fitting. The μ_i_ are the electric dipole moments, expressed as absolute values in Debye. σ represents the root-mean-square deviation of the fit, and *N* denotes the number of experimentally observed lines. Δ*E*_CCSD(T)_ is the relative electronic energy in kJ mol^–1^ at the DLPNO-CCSD­(T)/def2-TZVPP level of theory; Δ*E*_0_ and Δ*G* ^298K^ are the relative zero-point corrected energy and Gibbs energy of the conformers at 298 K in kJ mol^–1^ at the B3LYP-D3BJ/def2-TZVP level of theory. The angle (ϕ_BCD‑A_) and dihedral angles (∠ Acetyl and ∠ A-Ring) are in degrees.

b The standard error (1σ) is in parentheses in units of the last digit.

c The symbols + + (very intense) and – (weak) denote the intensity of the experimentally observed transitions in the A-symmetry state. Forbidden c-type transitions were detected for the E-symmetry state. See the main text for discussion.

Since **P** is a closed-shell molecule, no other fine structure effect is expected in the rotational spectra, except for that arising from the coupling between the internal and overall rotation. Consequently, we attributed the observed splitting to the coupling of the internal rotation of one of the three methyl groups at C18, C19, and C21 and the overall rotation, causing the occurrence of the A–E doublets observed in Figure 2.^37^ To identify the specific methyl rotor responsible for the splitting, we computed the necessary theoretical parameters, such as the V_3_ barrier, δ (the angle between the internal rotation axis and the principal axis z), and ε (the angle between the principal axis x and the projection of the internal rotation axis onto the xy-plane) for all of the conformers. Our analysis led us to pinpoint the C21 acetyl methyl top as the culprit, with a methyl rotation barrier of 1.9 kJ mol^–1^, as the other two methyl groups at C18 and C19 possessed significantly higher energy barriers, 9.6 and 10.2 kJ mol^–1^, respectively (see SI for more details).

Subsequently, the internal rotation of the identified conformer was analyzed by identifying the transitions belonging to the E-symmetry state. The measured A–E splittings (see Tables S2 in the Supporting Information) were utilized to determine the internal rotation barrier V_3_ using the internal axis method outlined by Woods,^39^ in a fitting using the rigid rotor Hamiltonian, performed using the XIAM program.^40^ The key parameters associated with the methyl rotor are summarized in Table 2 and Table S2 of the SI.

**Table 2:** Predicted Spectroscopic Parameters of the Methyl Rotor for the Six Most Stable Conformers of **P** at the B3LYP-D3BJ/def2-TZVP Level of Theory, along with the Experimental Fitted Values for the Observed Rotamer Using the XIAM Program

	theoretical						experimental
conformer	**I/G- 1**	**I/T 2**	**I/G+ 3**	**II/G- 4**	**II/T 5**	**II/G+ 6**	
V_3_ /kJ mol^–1^ ^a^	1.9	3.2	7.4	1.9	3.2	7.4	2.4425 (25)^b^
ε (rad)	0.489	2.421	1.018	0.308	2.173	0.757	0.3904 (15)
δ (rad)	1.411	0.521	2.417	1.447	0.600	0.651	1.42241 (51)

a V_3_ is the potential energy barrier of the methyl rotor, and ε and δ are derived from the position of the methyl rotor with respect to the principal inertial axes. The experimental values are from XIAM’s fitting of A- and E-symmetry states. The F_0_ value was fixed to the theoretical value (F_0_ = 158.97 GHz).

b The standard error (1σ) is in parentheses in units of the last digit.

The molecule **P** can exist in different forms determined by the A ring configuration and the arrangements of the acetyl group. To identify the detected rotamer, we compared the experimental values of the rotational constants with those predicted for the six conformers postulated theoretically. The experimental values matched those predicted for the three conformers of family I. However, the three conformers in family I exhibited similar rotational constants, making it challenging to identify the specific conformer based solely on this data. Initially, we relied on the energy criteria to tentatively assign the detected rotamer to the conformer as **I/G- 1**. The fact that very weak b-type transitions and no c-type transitions were detected for the A-symmetry state is consistent with their very low predicted value of the dipole moment value component, μ_b_ ≈ 0.6 D and μ_c_ ≈ 0.1 D, which supported our assignment. Note that the rotational transition selection rules derived from the rigid-body rotation considerations must be slightly modified when the methyl internal rotation is included. In the case of progesterone, despite the absence of an electric dipole moment along the *c*-axis of the principal inertia frame, a few c-type transitions in the E-symmetry state (E*) were experimentally observed. This effect results from the competition between the asymmetry-induced splitting and torsional-rotation interaction. As progesterone is a prolate molecule exhibiting b-type spectra, this interaction produces c-type transitions with considerable intensity in the E-symmetry state when the asymmetry splitting of the K_a_ doublets is comparable in magnitude with the internal rotation splitting.^41,42^ Fortunately, the experimentally fitted internal rotor parameters are highly valuable in the assignment. It is worth noting that the main difference between the conformers **I/G- 1**, **I/T 2** and **I/G+ 3** lies in the position of the acetyl group, which contains the methyl group responsible for the splitting of the rotational transitions and consequently affects the theoretical values of V_3_, ε and δ parameters (Table 2). After carefully examining these, it becomes evident that the experimental values align only with the theoretical values of **I/G- 1**. Based on all of this information, the conformer detected in the gas phase is unequivocally **I/G- 1**, predicted as the global minimum.

The **I/T 2** conformer, characterized by a high dipole moment (μ_a_ = 4.5 D) and relatively low energy (4.1 kJ mol^–1^ above the global minimum), poses an intriguing prospect for experimental detection, despite its absence in the spectra. To explore this, we performed a relaxed potential energy scan of the C17–C20 bond using the B3LYP-D3BJ/def2-TZVP theoretical method. The results (Figure 1 and SI Figure S1) predicted the interconversion barriers between the conformers **I/T 2** → **I/G- 1** as 4.4 kJ mol^–1^. This value aligns closely with the maximum energy gap traversable using Ne as a carrier gas, typically between 4 and 5 kJ mol^–1^.^43^ The **I/T 2** conformer, or a considerable portion thereof, will likely convert to the **I/G- 1** conformer during jet expansion. Thus, our rotational spectroscopy findings suggest that the predominant form of **P** in the gas phase comprises the **I/G- 1** conformer.

In the next stage, we will use the data collected in the gas phase to rationalize the **P** structure and its significance in a biologically active environment. To accomplish this, we have selected several complexes of progesterone with biomolecules that we believe are biologically relevant and provide adequate resolution for comparison to our findings. These complexes include the progesterone receptor (**PR**),^15^ the fragment antigen-binding (Fab) region of the anti-progesterone antibody (**Fab-DB3**),^44^ the cytochrome P450 17A1 hydrolase enzyme (**P450**)^45^ and the mineralocorticoid receptor (**MR**).^46^ Additionally, we have included the monomeric structure of pure **P** in the crystal form (**PCr**).^47,48^ We will examine the structure of **P** in the gas phase, in its crystalline form, and with complexes of the aforementioned biomolecules, aiming to gain insights into the structural behavior of progesterone in a biological environment.

To compare the **P** structure in the gas phase with other structures, the first step involves pairing the carbon atoms of the B, C, and D rings in each crystal structure with their counterparts in the gas phase, followed by superimposing (aligning) the structures (Figure 3). Once the molecules were aligned, we measured the root–mean–square deviation (RMSD) of the average distance between two sets of superimposed carbon atoms of the B, C, and D rings. This approach is essential for assessing **P**’s carbon skeleton in different environments. The low RMSD values (≪1 Å) obtained across the structures (Table 3) highlight the rigidity of this region of the molecule. Notably, this analysis confirms that only two flexible regions in the molecule require further examination: the acetyl side chain and the A ring.

**Figure 3:**
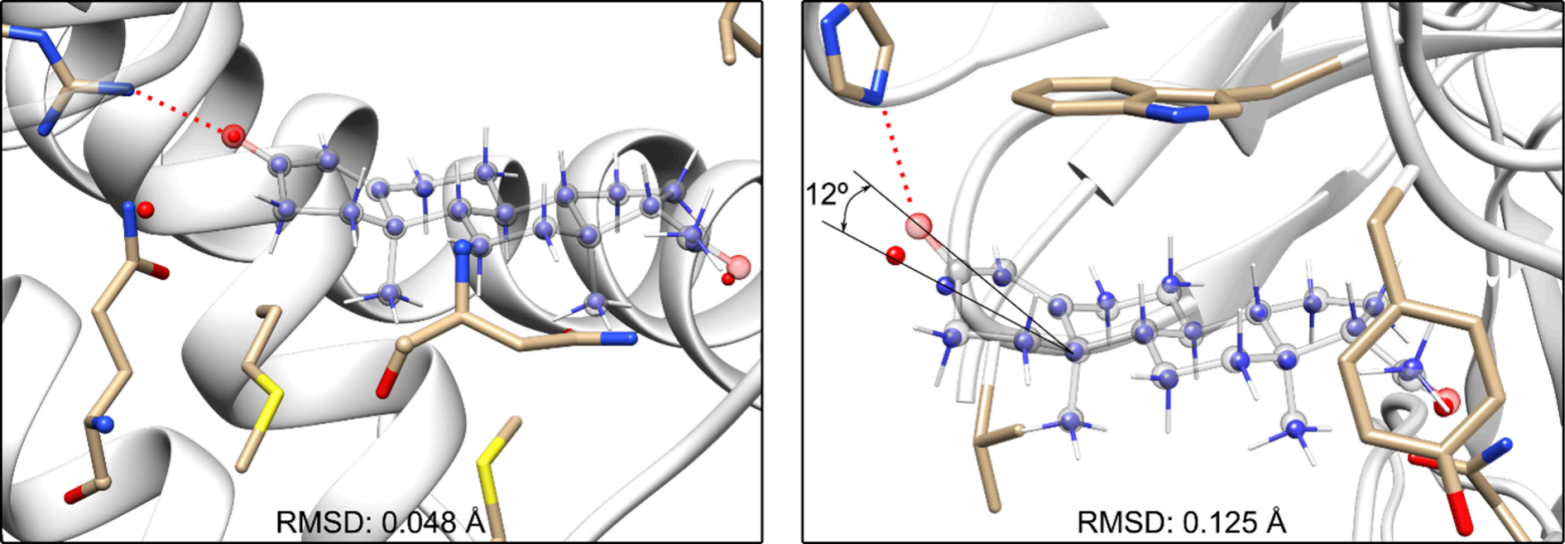
Structures superimposing the carbon atoms of the B, C, and D rings of the crystal structure of **P** interacting with a biomolecule (transparent) and the structure observed in the gas phase (solid balls for carbons and oxygen’s and solid sticks for hydrogens). The left figure corresponds to a structure of **P** interaction with progesterone receptor **PR**, and the right one corresponds to the structure of **P** interaction with **Fab-DB3**.

**Table 3:** Structural Parameters of Progesterone in Gas and Pure Crystal Phase and in the Crystal for Structures Combined with Biological Molecules

structure	**P** (**I/G- 1**)	**PCr**	**PR**	**Fab-DB3**	**P450**	**MR**
most similar conf.	n.a.	**I/G- 1**	**I/G- 1**	**I/G- 1**	**I/G+ 3** ^b^	**I/G- 1**
Δ*E* (kJ mol^–1^)	0.0	2.6	1.6	3.6	8.3	7.3
∠ acetyl	–89.0	–60.2	–69.2	–87.8	100.8	–74.2
ϕ _BCD‑A_	30.0	29.5	28.1	41.5	38.5	23.4
∠ A-Ring	–54.7	–56.0	–53.8	–62.3	–32.3	–36.8
RMSD (Å)^a^	0	0.078	0.048	0.125	0.144^b^	0.156

a RMSD defines the difference between the position of carbon atoms of B, C and D rings. Most similar represents the most similar conformer in the gas phase obtained from theoretical calculations. Δ*E* (kJ mol^–1^) is the energy difference of the crystal structure with respect to the most similar conformer in the gas phase (all the information in the text).

b The RMSD value of **P** in **P450** and the Δ*E* were obtained using the reference structure **I/G+3**.

The acetyl chain does not pose significant challenges, because the π-conjugation of the acetyl group ensures that the C17, C20, C21, and O23 atoms are in the same plane. As a result, the acetyl group acts as a rigid body, rotating over the C17–C20 bond. The potential energy surface has been previously presented (See Figure S1 in the Supporting Information). It is important to note that the rotation of the C17–C20 bond presents only one energetically unfavorable disposition, where the C20–C21 and C13–C17 bonds are eclipsed (≈ 0°). However, the acetyl group can adopt three stable orientations corresponding to the three conformers predicted by theoretical calculation. The detection of only the **I/G- 1** conformer in the gas phase, with the acetyl group arrangement in a “gauche-” disposition, suggests that interconversion between conformers with different acetyl group configurations (G–, T, G+) surely occurs without a significant energy barrier. This finding is consistent with the crystal structures of **P** in biological environments. None of the structures in Table 3 have an acetyl ≈0° arrangement, except in **P450**, in which all the structures of **P** monomers have a similar acetyl “gauche-” arrangement to that in the gas phase.

The A ring of the molecule presents a distinct challenge. We have used the aforementioned parameter ϕ_BCD‑A_) to describe **P**’s A-ring configuration. When comparing this parameter of structures of **P** in the gas phase and in the crystals, we observe three different tendencies: (i) In **PCr** and **PR**, the structure of **P** maintains almost the same configuration as that of **I/G- 1**. (ii) In **Fab-DB3** and **P450**, the A ring is positioned more perpendicularly to the B, C and D rings than in **I/G- 1**. It is numerically demonstrated by the increase in the value of the ϕ_BCD‑A_ angle. (iii) The final scenario is observed in **MR**, where **P** represents the structure with the A ring more parallel to B, C and D rings than in the gas phase (ϕ_BCD‑A_ ↓).

Initially, the results from the gas phase suggested that **P** is a relatively rigid molecule. However, upon examining the crystal structures, it became apparent that the A ring of the molecule may have some hidden flexibility. To investigate this behavior further and gain a deeper understanding, we compared the energy difference (Δ*E*) between the **P** structure in the crystals and the gas phase. To accomplish this, we utilized the structure of **P** in the crystals as a starting point, keeping the flexible dihedral angles of the **P** molecule fixed (including acetyl, ϕ_BCD‑A_, and A ring) in the crystalline structure, and optimized the position of the remaining atoms. Inherently, we found small energy differences between the gas phase and the crystal structures.

To understand this conformational behavior, we conducted two independent potential energy surface (PES) analyses: one focused on the ϕ_BCD‑A_ angle and another on the ∠ A-Ring dihedral angles (Figure 4). Two important observations can be made regarding these PES analyses. First, a high energy gap of 25 kJ mol^–1^ exists between the interconversion of conformers belonging to the I and II families when the interconversion occurs via rotation around the ∠ A-ring dihedral angle. Second, the ϕ _BCD‑A_ angle, which plays a major role in the flexibility of P, demonstrates greater flexibility than initially anticipated, with energy variations only exceeding 2 kJ mol^–1^ for angles below 20° and above 40°. Notably, the structures observed in the crystals indicate specific flexibility in binding of **P** with proteins. Figure 4 summarizes the range of ϕ_BCD‑A_ movement between 10–50°, revealing that it has minimal impact on the overall energy of the molecule.

**Figure 4:**
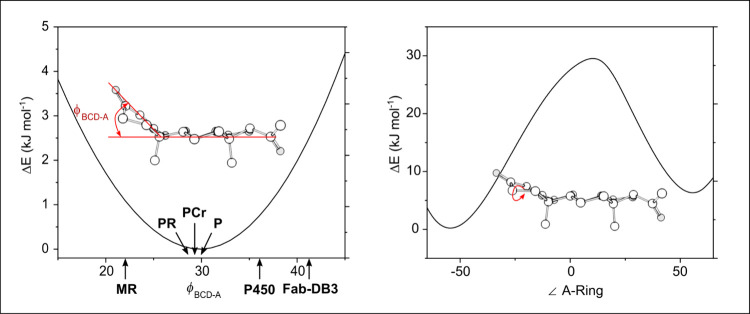
Relaxed PES of ϕ_BCD‑A_ and the ∠ A-Ring dihedral angle of the **I/G- 1** conformer of progesterone using the B3LYP-D3BJ/def2-TZVP level of theory.

In summary, we conducted a groundbreaking study on progesterone, a crucial hormone in the body. Taking advantage of rotational spectroscopy coupled with a laser ablation vaporization source in a supersonic expansion and quantum chemistry calculations, we elucidated the most stable conformer of **P**. The intrinsically preferred 3D shape of **P** in a free environment, along with bibliographic data of **P** structures and other progestogens complexed with ligand-binding domains, provided valuable insight into the significance of the conformation of these molecules in their biological activity.

The initial appearance of progesterone’s structure suggests rigidity, but the A ring is surprisingly flexible and capable of movement without significantly affecting the molecule’s energy. This is particularly interesting in **P** and **Ps** derivatives because their active biological sites have commonly conserved strong intermolecular interactions involving the A-ring part of these molecules.^49,50^ The flexibility of the progesterone receptor’s ligand-binding pocket has been previously analyzed;^51^ however, not much was known about the intrinsic molecular flexibility of the A-ring in progesterone and probably in other similar **Ps** molecules. Our results demonstrate that, for instance, the modification of the rigidity and dispositions of the A-ring could lead to compounds with very different affinities for **PR** and other proteins.

## Supplementary Material



## Notes

Detailed experimental and theoretical sections; theoretical coordinates of stable structures of progesterone (Table S1); PES scan of acetyl group within the family **I** and **II** conformers (Figure S1); theoretically predicted and experimentally fitted spectroscopic parameters (Table S2); figure of the crystal structures superimposed with the structure in the gas phase (Figure S2); and assigned rotational transitions of the I/G-1 conformer of progesterone (Tables S3, S4) (PDF)

## References

[1] Azeez J. M., Susmi T. R., Remadevi V., Ravindran V., Sasikumar Sujatha A., nair Sobha Ayswarya R., Sreeja S. (2021). New Insights into the
Functions of Progesterone Receptor (PR) Isoforms and Progesterone
Signaling. Am. J. Cancer Res..

[2] Kolatorova L., Vitku J., Suchopar J., Hill M., Parizek A. (2022). Progesterone:
A Steroid with Wide Range of Effects in Physiology as Well as Human
Medicine. IJMS.

[3] Conneely O. (2000). Progesterone
Receptors in Reproduction: Functional Impact of the A and B Isoforms. Steroids.

[4] Jacobsen B. M., Horwitz K. B. (2012). Progesterone Receptors,
Their Isoforms and Progesterone
Regulated Transcription. Mol. Cell. Endocrinol..

[5] Kastner P., Krust A., Turcotte B., Stropp U., Tora L., Gronemeyer H., Chambon P. (1990). Two Distinct Estrogen-Regulated
Promoters
Generate Transcripts Encoding the Two Functionally Different Human
Progesterone Receptor Forms A and B. EMBO Journal.

[6] Fagart J., Huyet J., Pinon G. M., Rochel M., Mayer C., Rafestin-Oblin M.-E. (2005). Crystal Structure of a Mutant Mineralocorticoid Receptor
Responsible for Hypertension. Nat. Struct Mol.
Biol..

[7] Jung C., Fernández-Dueñas V., Plata C., Garcia-Elias A., Ciruela F., Fernández-Fernández J. M., Valverde M. A. (2018). Functional Coupling of GABA _A/B_ Receptors
and the Channel TRPV4Mediates Rapid Progesterone Signaling in the
Oviduct. Sci. Signal..

[8] Crews D., Godwin J., Hartman V., Grammer M., Prediger E. A., Sheppherd R. (1996). Intrahypothalamic
Implantation of Progesterone in Castrated
Male Whiptail Lizards (*Cnemidophorus Inornatus*) Elicits
Courtship and Copulatory Behavior and Affects Androgen Receptor- and
Progesterone Receptor-mRNA Expression in the Brain. J. Neurosci..

[9] Gellersen B., Fernandes M. S., Brosens J. J. (2008). Non-Genomic Progesterone Actions
in Female Reproduction. Hum. Reprod. Update..

[10] Ogara M. F., Rodríguez-Seguí S. A., Marini M., Nacht A. S., Stortz M., Levi V., Presman D. M., Vicent G. P., Pecci A. (2019). The Glucocorticoid Receptor Interferes with Progesterone Receptor-Dependent
Genomic Regulation in Breast Cancer Cells. Nucleic
Acids Res..

[11] Mohammed H., Russell I. A., Stark R., Rueda O. M., Hickey T. E., Tarulli G. A., Serandour A. A., Birrell S. N., Bruna A., Saadi A., Menon S., Hadfield J., Pugh M., Raj G. V., Brown G. D., D’Santos C., Robinson J. L. L., Silva G., Launchbury R., Perou C. M., Stingl J., Caldas C., Tilley W. D., Carroll J. S. (2015). Progesterone Receptor Modulates ERα Action in
Breast Cancer. Nature.

[12] Pedroza D. A., Subramani R., Lakshmanaswamy R. (2020). Classical and Non-Classical Progesterone
Signaling in Breast Cancers. Cancers.

[13] An W., Lin H., Ma L., Zhang C., Zheng Y., Cheng Q., Ma C., Wu X., Zhang Z., Zhong Y., Wang M., He D., Yang Z., Du L., Feng S., Wang C., Yang F., Xiao P., Zhang P., Yu X., Sun J.-P. (2022). Progesterone Activates GPR126 to Promote Breast Cancer
Development via the Gi Pathway. Proc. Natl.
Acad. Sci. U.S.A..

[14] Hughes G. C. (2012). Progesterone
and Autoimmune Disease. Autoimmun. Rev..

[15] Williams S. P., Sigler P. B. (1998). Atomic Structure of Progesterone
Complexed with Its
Receptor. Nature.

[16] Ali S., Balachandran K., O’Malley B. (2020). 90 Years of Progesterone: Ninety
Years of Progesterone: The ‘Other’ Ovarian Hormone. J. Mol. Endocrinol..

[17] Alonso E. R., León I., Kolesniková L., Mata S., Alonso J. L. (2021). Unveiling
Five Naked Structures of Tartaric Acid. Angew.
Chem., Int. Ed..

[18] Steber A. L., Temelso B., Kisiel Z., Schnell M., Pérez C. (2023). Rotational
Dive into the Water Clusters on a Simple Sugar Substrate. Proc. Natl. Acad. Sci. U.S.A..

[19] Li W., Pérez C., Steber A. L., Schnell M., Lv D., Wang G., Zeng X., Zhou M. (2023). Evolution of Solute-Water
Interactions in the Benzaldehyde-(H2O)­1–6 Clusters by Rotational
Spectroscopy. J. Am. Chem. Soc..

[20] Hazrah A. S., Insausti A., Ma J., Al-Jabiri M. H., Jäger W., Xu Y. (2023). Wetting vs Droplet Aggregation: A
Broadband Rotational Spectroscopic Study of 3-Methylcatechol···Water
Clusters. Angew. Chem. Int. Ed.

[21] Insausti A., Ma J., Yang Q., Xie F., Xu Y. (2022). Rotational Spectroscopy
of 2-Furoic Acid and Its Dimer: Conformational Distribution and Double
Proton Tunneling. ChemPhysChem.

[22] Feng G., Favero L. B., Maris A., Vigorito A., Caminati W., Meyer R. (2012). Proton Transfer in
Homodimers of Carboxylic Acids: The Rotational
Spectrum of the Dimer of Acrylic Acid. J. Am.
Chem. Soc..

[23] Alonso J. L., Vaquero V., Peña I., López J. C., Mata S., Caminati W. (2013). All Five Forms of Cytosine Revealed
in the Gas Phase. Angew. Chem., Int. Ed..

[24] Favero L., Uriarte I., Spada L., Ecija P., Calabrese C., Caminati W., Cocinero E. J. (2016). Solving the Tautomeric Equilibrium
of Purine through Analysis of the Complex Hyperfine Structure of the
Four 14N Nuclei. J. Phys. Chem. Lett..

[25] Brown G. G., Dian B. C., Douglass K. O., Geyer S. M., Shipman S. T., Pate B. H. (2008). A Broadband Fourier Transform Microwave Spectrometer
Based on Chirped Pulse Excitation. Rev. Sci.
Instrum..

[26] Pinacho P., Caliebe S. V. M., Quesada-Moreno M. M., Zinn S., Schnell M. (2022). Unexpected
Discovery of Estrone in the Rotational Spectrum of Estradiol: A Systematic
Investigation of a CP-FTMW Spectrum. Phys. Chem.
Chem. Phys..

[27] Zinn S., Schnell M. (2018). Flexibility at the Fringes: Conformations of the Steroid
Hormone β -Estradiol. ChemPhysChem.

[28] Caliebe S. V. M., Pinacho P., Schnell M. (2022). Steroid Hormone Androsterone Observed
in the Gas Phase by Rotational Spectroscopy. J. Phys. Chem. Lett..

[29] León I., Alonso E. R., Mata S., Alonso J. L. (2021). Shape of Testosterone. J. Phys.
Chem. Lett..

[30] León I., Alonso E. R., Cabezas C., Mata S., Alonso J. L. (2019). Unveiling
the N→π* Interactions in Dipeptides. Commun. Chem..

[31] Schrödinger Release 2017-1, Maestro, Schrödinger, LLC, New York, NY, 2017.

[32] Becke A. D. (1993). Density-functional
Thermochemistry. III. The Role of Exact Exchange. J. Chem. Phys..

[33] Grimme S., Antony J., Ehrlich S., Krieg H. (2010). A Consistent and Accurate *Ab Initio* Parametrization
of Density Functional Dispersion
Correction (DFT-D) for the 94 Elements H-Pu. J. Chem. Phys..

[34] Grimme S., Ehrlich S., Goerigk L. (2011). Effect of the Damping Function in
Dispersion Corrected Density Functional Theory. J. Comput. Chem..

[35] Weigend F., Ahlrichs R. (2005). Balanced Basis Sets
of Split Valence, Triple Zeta Valence
and Quadruple Zeta Valence Quality for H to Rn: Design and Assessment
of Accuracy. Phys. Chem. Chem. Phys..

[36] Riplinger C., Neese F. (2013). An Efficient and near
Linear Scaling Pair Natural Orbital Based Local
Coupled Cluster Method. J. Chem. Phys..

[37] Gordy, W. ; Cook, R. L. Microwave Molecular Spectra; Wiley Interscience: New York, 1984.

[38] Pickett H. M. (1991). The Fitting
and Prediction of Vibration-Rotation Spectra with Spin Interactions. J. Mol. Spectrosc..

[39] Woods R. C. (1966). A General
Program for the Calculation of Internal Rotation Splittings in Microwave
Spectroscopy. J. Mol. Spectrosc..

[40] Hartwig H., Dreizler H. (1996). The Microwave Spectrum
of Trans-2,3-E-Dimethyloxirane
in Torsional Excited States. Z. Naturforsch..

[41] Scappini F., Dreizler H. (1981). Internal Rotation Spectrum
in the Ground State of Eis
Propionyl Fluoride. Z. Naturforsch..

[42] Dang N.-N., Pham H.-N., Kleiner I., Schwell M., Grabow J.-U., Nguyen H. V. L. (2022). Methyl Internal
Rotation in Fruit Esters: Chain-Length
Effect Observed in the Microwave Spectrum of Methyl Hexanoate. Molecules.

[43] Ruoff R. S., Klots T. D., Emilsson T., Gutowsky H. S. (1990). Relaxation
of Conformers
and Isomers in Seeded Supersonic Jets of Inert Gases. J. Chem. Phys..

[44] Arevalo J. H., Stura E. A., Taussig M. J., Wilson I. A. (1993). Three-Dimensional
Structure of an Anti-Steroid Fab′ and Progesterone-Fab′
Complex. J. Mol. Biol..

[45] Pallan P. S., Nagy L. D., Lei L., Gonzalez E., Kramlinger V. M., Azumaya C. M., Wawrzak Z., Waterman M. R., Guengerich F. P., Egli M. (2015). Structural and Kinetic
Basis of Steroid 17α,20-Lyase Activity
in Teleost Fish Cytochrome P450 17A1 and Its Absence in Cytochrome
P450 17A2. J. Biol. Chem..

[46] Fagart J., Huyet J., Pinon G. M., Rochel M., Mayer C., Rafestin-Oblin M.-E. (2005). Crystal
Structure of a Mutant Mineralocorticoid Receptor
Responsible for Hypertension. Nat. Struct Mol.
Biol..

[47] Bruhn J. F., Scapin G., Cheng A., Mercado B. Q., Waterman D. G., Ganesh T., Dallakyan S., Read B. N., Nieusma T., Lucier K. W., Mayer M. L., Chiang N. J., Poweleit N., McGilvray P. T., Wilson T. S., Mashore M., Hennessy C., Thomson S., Wang B., Potter C. S., Carragher B. (2021). Small Molecule
Microcrystal Electron Diffraction for the Pharmaceutical Industry-Lessons
Learned From Examining Over Fifty Samples. Front.
Mol. Biosci..

[48] Campsteyn H., Dupont L., Dideberg O. (1972). Structure
cristalline et moléculaire
de la progestérone, C21H30O2. Acta Crystallogr.
B Struct Crystallogr. Cryst. Chem..

[49] Khan S. H., Dube N., Sudhakar N., Fraser O., Villalona P., Braet S. M., Leedom S., Reilly E. R., Sivak J., Crittenden K., Okafor C. D. (2024). Ancient and Modern Mechanisms Compete
in Progesterone Receptor Activation. RSC Chem.
Biol..

[50] Madauss K. P., Deng S.-J., Austin R. J. H., Lambert M. H., McLay I., Pritchard J., Short S. A., Stewart E. L., Uings I. J., Williams S. P. (2004). Progesterone Receptor Ligand Binding Pocket Flexibility:
Crystal Structures of the Norethindrone and Mometasone Furoate Complexes. J. Med. Chem..

[51] Goswami D., Callaway C., Pascal B. D., Kumar R., Edwards D. P., Griffin P. R. (2014). Influence of Domain
Interactions on Conformational
Mobility of the Progesterone Receptor Detected by Hydrogen/Deuterium
Exchange Mass Spectrometry. Structure.

