# Supplementary material for: Determining the Molecular Shape of Progesterone: Insights from Laser Ablation Rotational Spectroscopy
Source: J Phys Chem Lett. 2025 Feb 27;16(9):2425–32. doi: 10.1021/acs.jpclett.4c03618 (PMC12131209; doi:10.1021/acs.jpclett.4c03618)
Supplement: Supplementary file 1 [file jz4c03618_si_001.pdf]

Supporting information of: Determining the Molecular Shape of Progesterone: Insights from Laser Ablation Rotational Spectroscopy

AUTHOR NAMES. Aran Insausti,[1,2] Elena R. Alonso,[3] Sofía Municio,[3] Iker León,[3] Lucie Kolesniková,[4] Santiago Mata [3]*

AUTHOR ADDRESS

[1] Departamento de Química-Física, Facultad de Ciencia y Tecnología, Universidad del País Vasco (UPV/EHU), 48940 Leioa, Spain;

[2] Instituto Biofisika (UPV/EHU, CSIC), University of the Basque Country, 48940 Leioa, Spain.

[3] Grupo de Espectrocopía Molecular (GEM), Edificio Quifima, Area de Química-Física, Laboratorios de Espectroscopia y Bioespectroscopia, Parque Científico UVa, Unidad Asociada CSIC, Universidad de Valladolid, 47011 Valladolid, Spain.

[4] Department of Analytical Chemistry, University of Chemistry and Technology, Technicka´ 5, 166 28 Prague 6, Czech Republic

AUTHOR INFORMATION

Corresponding Author

Elena R. Alonso:

Grupo de Espectrocopía Molecular (GEM), Edificio Quifima, Area de Química-Física, Laboratorios de Espectroscopia y Bioespectroscopia, Parque Científico UVa, Unidad Asociada CSIC, Universidad de Valladolid, 47011 Valladolid, Spain.

Email: elenarita.alonso@uva.es

Contents

Experimental details...............S3

Computational methods...............S4

Table S1. Theoretical coordinates of atoms of stable structures of progesterone obtained at B3LYP-D3BJ/def2-TZVP level of theory. S5-S7

Figure S1. Solid line represents the relaxation potential energy scan of acetyl group within the family **I** conformers and dashed line represent the same PES within the in the family **II** conformers...............S8

Table S2: Predicted spectroscopic parameters for the most stable conformers of **P**, **I/G- 1**, at B3LYP-D3BJ/def2-TZVP level of theory, along with the experimental fitted values for the observed rotamer...............S8

Figure S2: Crystal structures superimposed with the structures obtained experimentally in gas phase. S9

Experimental results...............S10

Table S3. Assigned rotational transitions of A-state of I/G- 1 conformer of progesterone using Pickett´s programS10-S12

Table S4. Assigned rotational transitions of A and E-states of I/G- 1 conformer of progesterone using Xiam program S12-S17

Bibliography...............S19

Experimental details:

We use a home-made **C**hirped **P**ulse **F**ourier **T**ransform **M**icro **W**ave spectrometer based on Pate´s apparatus, equipped with ultrafast (λ=355 nm and 35 ps) **L**aser **A**blation vaporization system (LA-CP-FTMW) to record the rotational spectra of pure **P**.[1,2] The solid sample of progesterone was prepared as follows: First we grind pure **P** powder; then we mix it with a small amount of glue, and we compress with hydraulic press to obtain a rod-shaped solid. Finally, the rod was dried in a desiccator for four weeks. In the experimental setup, the aforementioned rod is located in the motor. Under this system, the sample can move circularly providing a fresh sample for each laser shot and valve opening. The molecules were expanded in a vacuum chamber using pure Ne (10 bar) as carrier gas. Molecular excitation was carried out by generating 1.5 to 6.5 GHz up-chirp using a AWG (Tectronix 24 Gs/s) and amplifying the radiation using a pulsed solid-state power amplifier (250 W). The molecular rotational resonant emission was amplified using a low noise amplifier and digitalised with 50 Gs/s oscilloscope. After several experimental optimizations, 70k free induction decays were acquired and averaged. Laser ablation vaporization is a process that produces a sample that is not ejected, which obstructs gas expansion in the chamber in a short time. It does not allow us to average more FID. Obtained spectra can be seen in the Figure 2

Computational methodology:

The conformational landscape of progesterone was theoretically predicted using a two-step method combining molecular mechanics (MM) and quantum chemistry (QC). In the first step, all possible molecular configurations were evaluated using the Monte Carlo method based on atomic redistribution and energetic optimization through MMFFs force field molecular mechanics, implemented in the Maestro software (Schrödinger 2017).[3–6] Obtained conformers bellow 25 kJ mol^{-1} (six in total) were used in the next step. We also employed our chemical intuition to draw conformers that could represent stable structures.

In the second step, all structures from the first step were further optimized using density functional theory (DFT) calculations with the B3LYP-D3BJ method, employing the def2-TZVP basis sets.[7–13] The frequency calculations were also carried out at the same level of theory to ensure that the optimized structures corresponds to a local minima and to get each conformer's zero point correction energy. We used Gaussian 16 with Revision C.01 version to carry out all these calculations.[14] Finally, a single point electronic energy calculations of all the stable structures were done at DLPNO-CCSD(T)/def2-TZVPP level of theory,[15] with the resolution-of-identity (RIJCOSX) approximation,[16] by version 6.0 of Orca package.[17]

The relaxed electronic potential energy scans of the methyl rotor were carried out in 10° increments. For the $\phi_{BCD\text{-}A}$, the PES was obtained by varying the angle by 4° increments. Similarly, for the ∠ A-Ring, the dihedral angle was varied in 4° increments. These calculations were carried out using B3LYP-D3BJ method, employing the def2-TZVP basis sets and resolution-of-identity (RIJCOSX) approximation.

The structure overlap, the RMSD matching, and the drawing were performed using Chimera software.[18]

Table S1: Theoretical coordinates of atoms of stable structures of progesterone in Angstroms obtained at B3LYP-D3BJ/def2-TZVP level of theory.

I/G- 1			
**Atom**	**X**	**Y**	**Z**
C	5.03268	1.97842	-0.97402
C	4.79044	0.65382	-0.28843
O	5.53971	0.24614	0.57096
C	3.56706	-0.12726	-0.73181
C	2.55129	0.23720	1.56620
C	2.26312	0.21844	0.05787
C	-2.16983	0.55550	2.02002
C	-2.17540	0.41647	0.48245
O	-6.10266	0.31136	-1.04111
C	-4.99181	0.35039	-0.54517
C	3.70690	-1.66005	-0.57396
C	2.30214	-2.19334	-0.19532
C	1.38827	-0.97943	-0.36852
C	0.12801	1.56175	0.22372
C	1.54319	1.49731	-0.36613
C	-0.72395	0.32250	-0.09539
C	0.00438	-0.98905	0.26780
C	-2.22632	-2.15619	0.39467
C	-0.81644	-2.18991	-0.19190
C	-2.92852	-0.85778	0.12411
C	-2.87532	1.63854	-0.14734
C	-4.39237	1.62710	-0.00119
C	-4.17166	-0.85413	-0.38157
H	0.11732	-1.05394	1.35478
H	1.22716	-0.85581	-1.44868
H	-0.84880	0.29537	-1.18642
H	5.32867	1.79246	-2.01065
H	4.12016	2.57547	-1.01067
H	5.82400	2.52585	-0.46708
H	3.37140	0.12572	-1.77794
H	1.63543	0.25007	2.15353
H	3.13901	-0.62288	1.88085
H	3.12427	1.12665	1.83201
H	-3.18811	0.54693	2.40894
H	-1.62967	-0.25798	2.50202
H	-1.70472	1.49297	2.32301
H	4.44351	-1.88096	0.19578
H	4.06970	-2.10359	-1.50119
H	2.28290	-2.54512	0.83840
H	1.99883	-3.03161	-0.82157
H	-0.36428	2.45636	-0.15776
H	0.20036	1.69221	1.30444
H	1.48227	1.52646	-1.45922
H	2.09680	2.38648	-0.05408
H	-2.15411	-2.29103	1.48031
H	-2.82718	-2.98481	0.01821
H	-0.32330	-3.12057	0.09414
H	-0.87672	-2.18505	-1.28570
H	-2.63074	1.66238	-1.21367
H	-2.47488	2.55479	0.28705
H	-4.68093	1.69546	1.05310
H	-4.85364	2.47353	-0.50975
H	-4.65863	-1.78568	-0.64811

I/T 2			
**Atom**	**X**	**Y**	**Z**
C	6.08871	0.18020	-0.89877
C	4.77397	0.73641	-0.39533
O	4.71627	1.83309	0.11486
C	3.54929	-0.12957	-0.61202
C	2.49101	0.38292	1.62536
C	2.24433	0.25483	0.11231
C	-2.22072	0.69199	1.95795
C	-2.19772	0.44182	0.43460
O	-6.09871	0.19428	-1.14045
C	-4.99587	0.27874	-0.63197
C	3.73396	-1.64186	-0.29175
C	2.31303	-2.17076	0.03995
C	1.39007	-0.97966	-0.24145
C	0.10120	1.58879	0.12732
C	1.52915	1.49261	-0.42449
C	-0.73505	0.31914	-0.10911
C	-0.00614	-0.95781	0.36453
C	-2.23070	-2.13104	0.53329
C	-0.80961	-2.19516	-0.02301
C	-2.93593	-0.86076	0.15705
C	-2.89471	1.60910	-0.29433
C	-4.41401	1.59645	-0.17382
C	-4.17018	-0.90399	-0.36844
H	0.08453	-0.94197	1.45536
H	1.24929	-0.93997	-1.33075
H	-0.84012	0.20778	-1.19699
H	6.41942	-0.63044	-0.24477
H	5.97821	-0.24414	-1.89884
H	6.84196	0.96458	-0.90319
H	3.35670	-0.04570	-1.69111
H	1.55918	0.45390	2.18293
H	3.03889	-0.47172	2.02344
H	3.07591	1.27709	1.82893
H	-3.24570	0.70416	2.32887
H	-1.68418	-0.08050	2.50688
H	-1.76593	1.65197	2.19944
H	4.40335	-1.77578	0.55856
H	4.18243	-2.17392	-1.12986
H	2.24744	-2.47064	1.08760
H	2.05424	-3.04446	-0.55722
H	-0.39143	2.44419	-0.33506
H	0.14769	1.80717	1.19530
H	1.48946	1.43034	-1.51791
H	2.08730	2.39625	-0.17751
H	-2.17815	-2.18673	1.62700
H	-2.81911	-2.98925	0.20653
H	-0.31611	-3.09966	0.33796
H	-0.84925	-2.26886	-1.11534
H	-2.63112	1.55726	-1.35509
H	-2.50759	2.55731	0.07879
H	-4.72171	1.74034	0.86743
H	-4.87178	2.39952	-0.75125
H	-4.64614	-1.85633	-0.57477

I/G+ 3			
Atom	X	Y	Z
C	-5.85794	-0.25902	0.47056
C	-4.72681	-0.77755	-0.38620
O	-4.73233	-1.92046	-0.79107
C	-3.56202	0.13266	-0.73724
C	-2.53933	-0.26414	1.54689
C	-2.25730	-0.21524	0.03666
C	2.15305	-0.58410	2.01029
C	2.16863	-0.41792	0.47544
O	6.10979	-0.28476	-1.01067
C	4.99370	-0.33363	-0.52738
C	-3.72212	1.66071	-0.53685
C	-2.30839	2.20075	-0.18655
C	-1.38947	0.99028	-0.36999
C	-0.13143	-1.56459	0.17412
C	-1.54595	-1.48690	-0.41551
C	0.72074	-0.31522	-0.11031
C	-0.00902	0.99144	0.27293
C	2.22045	2.15635	0.43247
C	0.81437	2.19950	-0.16231
C	2.92433	0.86230	0.14514
C	2.87330	-1.62844	-0.17107
C	4.38922	-1.62017	-0.01324
C	4.17158	0.86772	-0.35025
H	-0.12732	1.03776	1.36061
H	-1.22401	0.87956	-1.45027
H	0.85313	-0.26501	-1.19955
H	-5.48278	0.16915	1.40181
H	-6.38824	0.53975	-0.05425
H	-6.54697	-1.07203	0.68641
H	-3.35528	-0.07425	-1.79018
H	-1.62563	-0.35455	2.13029
H	-3.05838	0.62761	1.89815
H	-3.16017	-1.12980	1.78469
H	3.16881	-0.58474	2.40590
H	1.61169	0.22213	2.50324
H	1.68374	-1.52566	2.29321
H	-4.42444	1.88903	0.26279
H	-4.11965	2.12562	-1.43903
H	-2.27384	2.55951	0.84446
H	-2.02135	3.03798	-0.82157
H	0.36280	-2.44627	-0.23368
H	-0.19964	-1.72829	1.25080
H	-1.48553	-1.48498	-1.50809
H	-2.11737	-2.37477	-0.14021
H	2.14144	2.27346	1.51975
H	2.82371	2.99095	0.07336
H	0.31951	3.12605	0.13499
H	0.88147	2.21149	-1.25562
H	2.63650	-1.63285	-1.23932
H	2.46903	-2.55211	0.24327
H	4.67002	-1.70926	1.04164
H	4.85406	-2.45683	-0.53454
H	4.66059	1.80379	-0.59660

II/G- 4			
Atom	X	Y	Z
C	4.82358	2.13331	-0.82401
C	4.63366	0.69997	-0.38484
O	5.45802	0.14036	0.30319
C	3.36403	0.00957	-0.84854
C	2.57937	-0.08413	1.56350
C	2.14637	0.18774	0.11521
C	-2.09866	-0.02638	2.45296
C	-2.23827	0.24636	0.94182
O	-5.44863	0.73800	-1.74016
C	-4.48070	0.60799	-1.01418
C	3.50023	-1.52420	-1.00138
C	2.13277	-2.13013	-0.59596
C	1.22023	-0.91198	-0.44974
C	0.04996	1.46548	0.73485
C	1.40885	1.52264	0.02236
C	-0.83854	0.31500	0.24122
C	-0.09698	-1.04386	0.30438
C	-2.34334	-2.24426	0.41325
C	-0.95337	-2.18627	-0.24494
C	-2.97463	-0.89554	0.26331
C	-3.04925	1.54904	0.80496
C	-3.62624	1.78170	-0.58439
C	-4.01380	-0.70906	-0.56561
H	0.12804	-1.27381	1.34940
H	0.96120	-0.58705	-1.46713
H	-1.03858	0.49873	-0.82175
H	5.01464	2.15214	-1.90100
H	3.91921	2.72008	-0.65565
H	5.66714	2.57869	-0.30161
H	3.07121	0.45710	-1.80279
H	3.17675	-0.98954	1.64989
H	3.19371	0.73705	1.93520
H	1.72514	-0.18109	2.23050
H	-3.07464	-0.25778	2.88244
H	-1.42865	-0.85119	2.68323
H	-1.71885	0.86191	2.95867
H	4.30576	-1.88338	-0.36421
H	3.76521	-1.77923	-2.02744
H	2.20954	-2.67026	0.35004
H	1.76110	-2.83780	-1.33621
H	-0.45808	2.41763	0.57965
H	0.20831	1.37677	1.81128
H	1.25086	1.77011	-1.03279
H	2.00165	2.33300	0.45387
H	-2.23591	-2.51860	1.46527
H	-2.95701	-3.00881	-0.06388
H	-0.43933	-3.13878	-0.10387
H	-1.08486	-2.05040	-1.32354
H	-2.44694	2.40044	1.12076
H	-3.88178	1.49074	1.51225
H	-4.22963	2.68810	-0.62898
H	-2.82825	1.89145	-1.32698
H	-4.50884	-1.55776	-1.02476

II/T 5			
Atom	X	Y	Z
C	5.86150	0.29193	-1.19308
C	4.61063	0.76531	-0.48428
O	4.62203	1.76617	0.19725
C	3.35447	-0.04962	-0.71859
C	2.51973	0.09831	1.66160
C	2.12991	0.21860	0.17819
C	-2.16559	0.22937	2.42062
C	-2.26985	0.33952	0.88624
O	-5.41392	0.52910	-1.91059
C	-4.46429	0.48178	-1.15079
C	3.53892	-1.59463	-0.66200
C	2.14566	-2.16417	-0.28302
C	1.22298	-0.94037	-0.28887
C	0.01937	1.54544	0.60110
C	1.39567	1.53344	-0.07784
C	-0.85354	0.34164	0.21566
C	-0.11184	-1.00108	0.44099
C	-2.35763	-2.19370	0.62199
C	-0.95249	-2.19847	-0.00561
C	-2.98744	-0.87089	0.31544
C	-3.08054	1.61634	0.59330
C	-3.62350	1.69820	-0.82652
C	-4.00631	-0.77816	-0.55364
H	0.08796	-1.11795	1.50954
H	0.98720	-0.72894	-1.34125
H	-1.02877	0.40815	-0.86541
H	6.23662	-0.61417	-0.71091
H	5.64980	0.03367	-2.23273
H	6.62575	1.06408	-1.14456
H	3.06419	0.20715	-1.74732
H	3.07607	-0.81695	1.86566
H	3.14760	0.93985	1.94552
H	1.64668	0.09449	2.31117
H	-1.79788	1.16760	2.83698
H	-3.15113	0.04170	2.84978
H	-1.50013	-0.56384	2.75273
H	4.28308	-1.86577	0.08747
H	3.89538	-1.98741	-1.61345
H	2.17271	-2.62334	0.70699
H	1.81614	-2.93322	-0.98080
H	-0.48955	2.47052	0.32898
H	0.14913	1.57810	1.68462
H	1.26143	1.65757	-1.15842
H	1.99197	2.37869	0.26703
H	-2.27487	-2.35480	1.69939
H	-2.95837	-3.00696	0.21379
H	-0.44049	-3.12946	0.24596
H	-1.05889	-2.17634	-1.09533
H	-2.48758	2.49873	0.83157
H	-3.93020	1.62882	1.28238
H	-4.22733	2.59207	-0.98158
H	-2.80726	1.73278	-1.55645
H	-4.48817	-1.67296	-0.93234

II/G+ 6			
Atom	X	Y	Z
C	5.76741	-0.16985	-0.16835
C	4.56482	-0.82851	0.46574
O	4.54583	-2.02547	0.65835
C	3.35881	0.00901	0.85693
C	2.57052	0.02831	-1.55031
C	2.14054	-0.19110	-0.09077
C	-2.07973	-0.05071	-2.45320
C	-2.22999	-0.27087	-0.93450
O	-5.46333	-0.66504	1.73587
C	-4.48816	-0.56205	1.01520
C	3.51610	1.54807	0.94737
C	2.13789	2.14743	0.55433
C	1.22087	0.92773	0.43713
C	0.05309	-1.49190	-0.66511
C	1.41005	-1.52319	0.05242
C	-0.83476	-0.31853	-0.22265
C	-0.09142	1.03816	-0.32835
C	-2.33509	2.23687	-0.49220
C	-0.94987	2.19909	0.17700
C	-2.96943	0.89459	-0.30112
C	-3.04402	-1.56695	-0.75869
C	-3.63163	-1.75049	0.63365
C	-4.01500	0.73842	0.52589
H	0.14009	1.23242	-1.37947
H	0.95638	0.63208	1.46139
H	-1.04246	-0.46196	0.84496
H	5.47938	0.42097	-1.03968
H	6.23365	0.51856	0.54113
H	6.48459	-0.93415	-0.45796
H	3.05387	-0.38131	1.83102
H	3.10324	0.96928	-1.68721
H	3.22937	-0.78037	-1.87156
H	1.72096	0.03782	-2.22965
H	-3.05258	0.16545	-2.89755
H	-1.40809	0.76589	-2.70725
H	-1.69608	-0.95602	-2.92454
H	4.29337	1.91236	0.27813
H	3.81186	1.84370	1.95394
H	2.20196	2.67665	-0.39885
H	1.77854	2.86565	1.29030
H	-0.45843	-2.43422	-0.46793
H	0.20877	-1.45291	-1.74515
H	1.25078	-1.72292	1.11647
H	2.01857	-2.34561	-0.32688
H	-2.22026	2.47541	-1.55220
H	-2.95090	3.01783	-0.04529
H	-0.43374	3.14638	0.00872
H	-1.08895	2.09840	1.25848
H	-2.44021	-2.42920	-1.03977
H	-3.87131	-1.53229	-1.47374
H	-4.23587	-2.65456	0.70537
H	-2.83911	-1.83479	1.38531
H	-4.51183	1.60307	0.95224

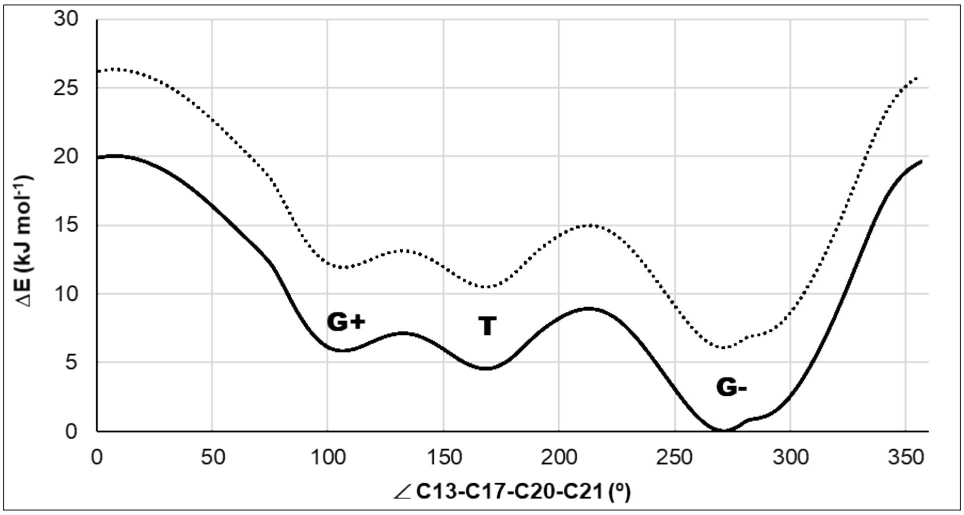

Figure S1. Solid line represents the relaxation potential energy scan of acetyl group within the family **I** conformers and dashed line represent the same PES within the in the family **II** conformers.

Table S2: Predicted spectroscopic parameters for the most stable conformers of **P**, **I/G- 1**, at B3LYP-D3BJ/def2-TZVP level of theory, along with the experimental fitted values for the observed rotamer.

	**Theoretical**	**Experimental**	
Conformer	**I/G- 1**	***A-symmetry (Pickett)***	***A & E-symmetries (Xiam)***
A (MHz)	681	676.30846 (41)[a]	676.30593 (44)
B (MHz)	131	131.250945 (71)	131.246928 (70)
C (MHz)	121	120.446229 (49)	120.445620 (55)
V_3 (kJ mol^{-1})	1.9	n.a.	2.4426 (27)
[b]ε (rad)	0.489	n.a.	0.3907 (17)
δ (rad)	1.411	n.a.	1.42242 (57)
[c]Δ_{Km} (kHz)[d]		n.a.	-67.1 (25)
δ_m (kHz)		n.a.	-6.05 (45)
σ (kHz)		10.6	12.3
N (exp lines)		121	235

[a] The standard error (1σ) is in parentheses in units of the last digit.
[b] ε, and δ angles are derived from the position of the methyl rotor respect to the principal inertia axes.
[c] Δ_{Km} and δ_m are the empirical internal rotation-overall rotation distortion operator defined in equation (6) of reference 19.

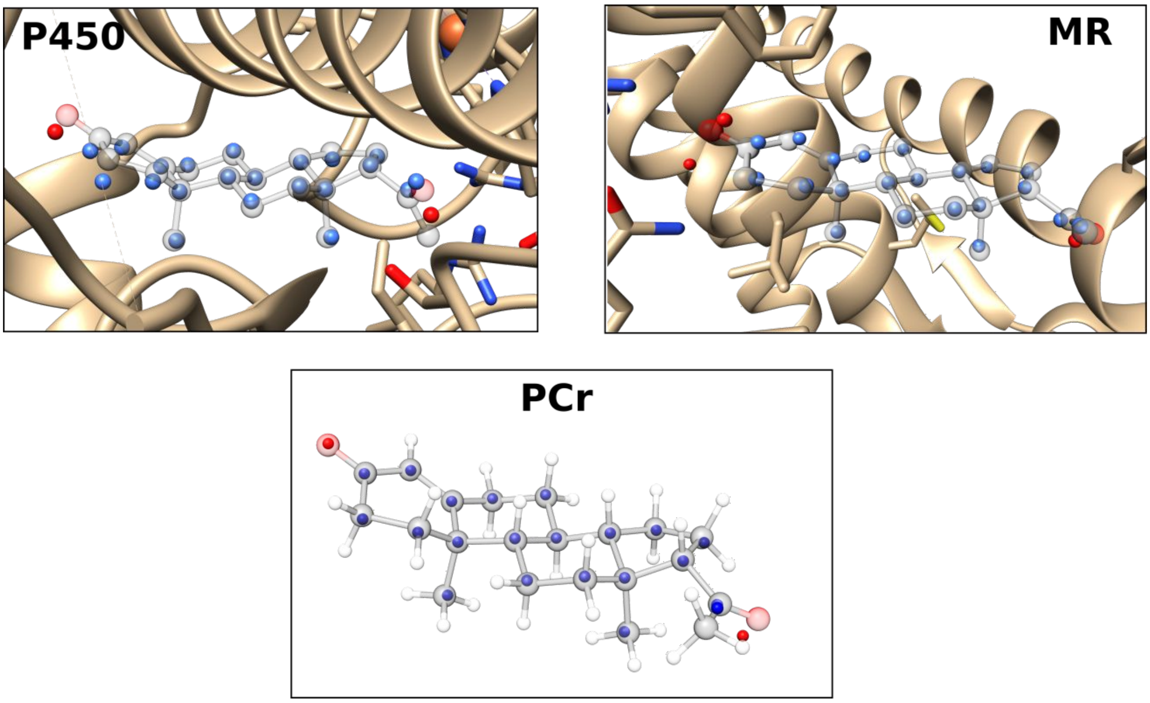

Figure S2: Crystal structures superimposed with the structures obtained experimentally in gas phase. The structures of the crystal have been obtained from References: 20–22

Experimental results

Table S3. Assigned rotational transitions of A-symmetry of **I/G- 1** conformer of progesterone using *Pickett´*s program.

J'	K_a'	K_c'		J''	K_a''	K_c''	ν_{Exp} (MHz)	Δν (kHz)
7	2	6	←	6	2	5	1760.651	6.8
7	2	5	←	6	2	4	1769.445	6.8
8	1	8	←	7	1	7	1967.384	25.7
8	2	7	←	7	2	6	2011.637	1.8
8	2	6	←	7	2	5	2024.715	4.4
9	0	9	←	8	0	8	2246.827	9.5
9	2	8	←	8	2	7	2262.419	4.1
9	3	6	←	8	3	5	2268.276	3.7
9	2	7	←	8	2	6	2280.870	6.5
8	1	8	←	7	0	7	2382.123	13.8
10	0	10	←	9	0	9	2492.029	17.9
10	2	9	←	9	2	8	2512.942	-16.8
10	3	8	←	9	3	7	2519.997	7.7
10	3	7	←	9	3	6	2521.141	1.1
10	2	8	←	9	2	7	2537.910	9.5
9	1	9	←	8	0	8	2594.006	5.0
11	0	11	←	10	0	10	2736.099	3.7
11	3	9	←	10	3	8	2772.474	6.6
11	3	8	←	10	3	7	2774.335	5.7
10	1	10	←	9	0	9	2804.342	11.4
11	1	10	←	10	1	9	2818.597	-10.0
12	1	12	←	11	1	11	2945.803	8.0
12	0	12	←	11	0	11	2979.145	6.6
11	1	11	←	10	0	10	3013.927	-8.7
12	3	10	←	11	3	9	3025.024	5.5
12	3	9	←	11	3	8	3027.906	5.8
12	1	11	←	11	1	10	3072.585	16.0
6	2	5	←	5	1	4	3080.472	3.3
13	1	13	←	12	1	12	3189.683	2.7
13	0	13	←	12	0	12	3221.261	5.5
12	1	12	←	11	0	11	3223.639	3.7
13	3	11	←	12	3	10	3277.636	12.4
13	3	10	←	12	3	9	3281.926	7.4
7	2	6	←	6	1	5	3299.826	-2.7
13	1	12	←	12	1	11	3325.844	-0.8
14	1	14	←	13	1	13	3433.277	1.2
13	1	13	←	12	0	12	3434.182	5.1
14	0	14	←	13	0	13	3462.596	1.6
3	3	1	←	2	2	0	3507.297	-33.9
3	3	0	←	2	2	1	3507.503	11.6
14	3	12	←	13	3	11	3530.258	-2.0

14	3	11	←	13	3	10	3536.478	22.2
14	1	13	←	13	1	12	3578.347	4.8
14	1	14	←	13	0	13	3646.219	21.6
15	1	15	←	14	1	14	3676.590	0.3
15	0	15	←	14	0	14	3703.324	2.1
4	3	2	←	3	2	1	3758.733	27.6
4	3	1	←	3	2	2	3759.508	-3.2
15	3	13	←	14	3	12	3782.910	10.2
15	3	12	←	14	3	11	3791.609	22.4
15	1	14	←	14	1	13	3829.971	4.8
15	1	15	←	14	0	14	3860.199	5.8
16	1	16	←	15	1	15	3919.634	0.4
10	2	9	←	9	1	8	3926.940	5.6
16	0	16	←	15	0	15	3943.607	1.5
5	3	3	←	4	2	2	4009.477	7.3
5	3	2	←	4	2	3	4011.891	-3.1
16	3	14	←	15	3	13	4035.514	4.4
16	3	13	←	15	3	12	4047.395	10.3
16	1	15	←	15	1	14	4080.616	-4.9
11	2	10	←	10	1	9	4126.117	-15.5
17	1	17	←	16	1	16	4162.438	15.2
17	0	17	←	16	0	16	4183.599	1.0
6	3	4	←	5	2	3	4259.209	25.5
6	3	3	←	5	2	4	4264.865	4.8
17	3	14	←	16	3	13	4303.918	-3.2
17	1	16	←	16	1	15	4330.225	7.8
17	2	15	←	16	2	14	4352.608	29.1
18	1	18	←	17	1	17	4404.971	-3.1
18	0	18	←	17	0	17	4423.426	-3.6
7	3	5	←	6	2	4	4507.299	0.0
7	3	4	←	6	2	5	4518.693	3.8
18	3	15	←	17	3	14	4561.263	7.7
18	1	17	←	17	1	16	4578.672	-0.7
18	2	16	←	17	2	15	4611.880	5.0
19	1	19	←	18	1	18	4647.307	1.6
19	0	19	←	18	0	18	4663.200	-0.5
8	3	5	←	7	2	6	4773.719	0.8
19	3	16	←	18	3	15	4819.412	-15.8
19	1	18	←	18	1	17	4825.925	1.3
4	4	1	←	3	3	0	4860.027	-4.0
4	4	0	←	3	3	1	4860.027	-5.4
19	2	17	←	18	2	16	4870.633	-26.6
20	1	20	←	19	1	19	4889.432	-5.0
20	0	20	←	19	0	19	4902.979	-5.3
9	3	6	←	8	2	7	5030.369	13.7
20	4	17	←	19	4	16	5047.512	-1.3

20	4	16	←	19	4	15	5050.151	5.3
20	1	19	←	19	1	18	5071.929	-2.3
20	3	17	←	19	3	16	5078.454	-0.8
5	4	2	←	4	3	1	5111.764	4.2
5	4	1	←	4	3	2	5111.764	-6.0
20	2	18	←	19	2	17	5128.804	1.6
21	1	21	←	20	1	20	5131.381	-7.2
21	0	21	←	20	0	20	5142.826	-3.3
21	4	18	←	20	4	17	5301.023	-8.9
21	4	17	←	20	4	16	5304.714	-1.2
21	1	20	←	20	1	19	5316.693	-0.3
21	3	18	←	20	3	17	5338.320	4.8
6	4	3	←	5	3	2	5363.440	4.7
6	4	3	←	5	3	2	5363.440	4.7
22	1	22	←	21	1	21	5373.164	-15.5
22	0	22	←	21	0	21	5382.760	-4.6
22	4	19	←	21	4	18	5554.646	6.6
22	1	21	←	21	1	20	5560.238	-5.1
23	0	23	←	22	0	22	5622.799	-3.6
23	1	22	←	22	1	21	5802.642	-15.3
23	4	20	←	22	4	19	5808.319	2.4
23	4	19	←	22	4	18	5815.154	-7.5
24	1	24	←	23	1	23	5856.346	-9.9
23	3	20	←	22	3	19	5860.238	-21.0
8	4	5	←	7	3	4	5866.401	-3.1
8	4	4	←	7	3	5	5866.710	-7.4
24	1	23	←	23	1	22	6044.045	-4.3
24	4	21	←	23	4	20	6062.032	-8.5
24	4	20	←	23	4	19	6071.142	-11.2
25	1	25	←	24	1	24	6097.762	-13.0
25	0	25	←	24	0	24	6103.181	-14.8
9	4	6	←	8	3	5	6117.522	-7.4
9	4	5	←	8	3	6	6118.196	-22.7
24	3	21	←	23	3	20	6122.087	-7.9

Table S4. Assigned rotational transitions of A and E-symmetries of **I/G- 1** conformer of progesterone using Xiam program. E* represents the c-type transitions of the E-symmetry state (details provided in the main text).

J'	K_a'	K_c'		J''	K_a''	K_c''	State	ν_{Exp} (MHz)	Δν (kHz)
7	2	6	←	6	2	5	A	1760.650	8.7
7	2	6	←	6	2	5	E	1761.802	15.2
7	2	5	←	6	2	4	E	1768.237	2.2
7	2	5	←	6	2	4	A	1769.445	8.9
8	1	8	←	7	1	7	A	1967.384	27.3
8	2	7	←	7	2	6	A	2011.637	3.9
8	2	7	←	7	2	6	E	2012.392	0.5
8	2	6	←	7	2	5	E	2023.889	2.8
8	2	6	←	7	2	5	A	2024.715	6.9
9	0	9	←	8	0	8	E	2246.585	-8.9
9	0	9	←	8	0	8	A	2246.827	11.5
9	2	8	←	8	2	7	A	2262.419	6.5
9	2	8	←	8	2	7	E	2262.863	4.3
9	3	6	←	8	3	5	E	2267.882	-9.8
9	3	6	←	8	3	5	A	2268.276	6.3
9	2	7	←	8	2	6	E	2280.345	1.2
9	2	7	←	8	2	6	A	2280.870	9.4
8	1	8	←	7	0	7	A	2382.123	13.5
8	1	8	←	7	0	7	E	2383.043	19.2
10	0	10	←	9	0	9	E	2491.757	-9.7
10	0	10	←	9	0	9	A	2492.029	20.0
10	2	9	←	9	2	8	A	2512.942	-14.2
10	2	9	←	9	2	8	E	2513.215	9.3
10	3	8	←	9	3	7	A	2519.997	10.6
10	3	8	←	9	3	7	E	2520.519	59.0
10	3	7	←	9	3	6	E	2520.519	-29.3
10	3	7	←	9	3	6	A	2521.141	4.0
10	2	8	←	9	2	7	E	2537.585	13.0
10	2	8	←	9	2	7	A	2537.910	12.9
9	1	9	←	8	0	8	A	2594.006	4.6
9	1	9	←	8	0	8	E	2595.192	-2.4
11	0	11	←	10	0	10	E	2735.819	4.7
11	0	11	←	10	0	10	A	2736.099	5.9
11	3	9	←	10	3	8	A	2772.474	9.7
11	3	9	←	10	3	8	E	2773.188	-4.8
11	3	8	←	10	3	7	E	2773.473	2.6
11	3	8	←	10	3	7	A	2774.335	9.0
10	1	10	←	9	0	9	A	2804.342	10.9
10	1	10	←	9	0	9	E	2805.863	7.7
11	1	10	←	10	1	9	E	2818.395	9.2

11	1	10	←	10	1	9	A	2818.597	-6.5
12	1	12	←	11	1	11	A	2945.803	10.3
12	1	12	←	11	1	11	E	2946.044	3.8
12	0	12	←	11	0	11	E	2978.801	0.5
12	0	12	←	11	0	11	A	2979.145	8.9
11	1	11	←	10	0	10	A	3013.927	-9.4
12	3	10	←	11	3	9	A	3025.024	8.9
12	3	10	←	11	3	9	E	3026.019	1.9
12	3	9	←	11	3	8	E	3026.759	3.5
12	3	9	←	11	3	8	A	3027.906	9.3
12	1	11	←	11	1	10	E	3072.293	-8.5
12	1	11	←	11	1	10	A	3072.585	19.9
6	2	5	←	5	1	4	A	3080.472	3.2
13	1	13	←	12	1	12	A	3189.683	5.1
13	1	13	←	12	1	12	E	3190.023	-11.0
13	0	13	←	12	0	12	E	3220.831	2.9
13	0	13	←	12	0	12	A	3221.261	7.8
12	1	12	←	11	0	11	A	3223.639	3.1
13	3	11	←	12	3	10	A	3277.636	16.1
13	3	11	←	12	3	10	E	3278.839	6.5
13	3	10	←	12	3	9	E	3280.552	2.5
13	3	10	←	12	3	9	A	3281.926	11.3
7	2	6	←	6	1	5	E	3297.255	16.1
7	2	6	←	6	1	5	A	3299.826	-3.1
13	1	12	←	12	1	11	E	3325.542	5.0
13	1	12	←	12	1	11	A	3325.844	3.2
14	1	14	←	13	1	13	A	3433.277	3.7
14	1	14	←	13	1	13	E	3433.768	-7.3
13	1	13	←	12	0	12	A	3434.182	4.5
13	1	13	←	12	0	12	E	3437.320	-11.8
14	0	14	←	13	0	13	E	3462.040	1.1
14	0	14	←	13	0	13	A	3462.596	4.0
3	3	1	←	2	2	1	E*	3503.785	-1.5
3	3	1	←	2	2	0	A	3507.297	-34.4
3	3	0	←	2	2	1	A	3507.503	11.2
3	3	0	←	2	2	0	E*	3510.809	-10.9
14	3	12	←	13	3	11	A	3530.258	1.9
14	3	12	←	13	3	11	E	3531.527	5.3
14	3	11	←	13	3	10	E	3535.036	15.1
14	3	11	←	13	3	10	A	3536.478	26.5
14	1	13	←	13	1	12	E	3578.003	0.7
14	1	13	←	13	1	12	A	3578.347	9.0
14	1	14	←	13	0	13	A	3646.219	21.2
14	1	14	←	13	0	13	E	3650.274	-4.2
15	1	15	←	14	1	14	A	3676.590	2.9
15	1	15	←	14	1	14	E	3677.281	-1.1

15	0	15	←	14	0	14	E	3702.585	-2.7
15	0	15	←	14	0	14	A	3703.324	4.7
4	3	2	←	3	2	2	E*	3755.455	-29.7
4	3	2	←	3	2	1	A	3758.733	27.4
4	3	1	←	3	2	2	A	3759.508	-3.4
4	3	1	←	3	2	1	E*	3762.486	-10.9
15	3	13	←	14	3	12	A	3782.910	14.5
15	3	13	←	14	3	12	E	3784.031	4.6
15	3	12	←	14	3	11	E	3790.282	7.8
15	3	12	←	14	3	11	A	3791.609	27.1
15	1	14	←	14	1	13	E	3829.604	-0.6
15	1	14	←	14	1	13	A	3829.971	9.2
15	1	15	←	14	0	14	A	3860.199	5.5
15	1	15	←	14	0	14	E	3865.525	3.2
16	1	16	←	15	1	15	A	3919.634	3.2
16	1	16	←	15	1	15	E	3920.576	1.5
10	2	9	←	9	1	8	E	3926.055	12.2
10	2	9	←	9	1	8	A	3926.940	4.4
16	0	16	←	15	0	15	E	3942.633	0.5
16	0	16	←	15	0	15	A	3943.607	4.1
5	3	3	←	4	2	3	E*	4007.140	6.2
5	3	3	←	4	2	2	A	4009.477	7.4
5	3	2	←	4	2	3	A	4011.891	-3.0
5	3	2	←	4	2	2	E*	4013.974	3.6
16	3	14	←	15	3	13	A	4035.514	8.9
16	3	14	←	15	3	13	E	4036.390	3.5
16	3	13	←	15	3	12	E	4046.308	-1.3
16	3	13	←	15	3	12	A	4047.395	15.3
16	1	15	←	15	1	14	E	4080.260	12.7
16	1	15	←	15	1	14	A	4080.616	-0.4
11	2	10	←	10	1	9	E	4125.566	24.9
11	2	10	←	10	1	9	A	4126.117	-17.1
17	1	17	←	16	1	16	A	4162.438	18.1
17	1	17	←	16	1	16	E	4163.679	2.4
17	0	17	←	16	0	16	E	4182.317	-2.5
17	0	17	←	16	0	16	A	4183.599	3.7
6	3	4	←	5	2	4	E*	4258.893	4.9
6	3	4	←	5	2	3	A	4259.209	25.9
6	3	3	←	5	2	4	A	4264.865	5.3
6	3	3	←	5	2	3	E*	4264.865	-5.7
17	3	14	←	16	3	13	E	4303.099	5.0
17	3	14	←	16	3	13	A	4303.918	2.2
17	1	16	←	16	1	15	E	4329.840	-0.3
17	1	16	←	16	1	15	A	4330.225	12.5
17	2	15	←	16	2	14	E	4352.380	-36.1
17	2	15	←	16	2	14	A	4352.608	35.2

18	1	18	←	17	1	17	A	4404.971	-0.2
18	1	18	←	17	1	17	E	4406.600	-4.0
18	0	18	←	17	0	17	E	4421.776	-2.0
18	0	18	←	17	0	17	A	4423.426	-0.9
7	3	5	←	6	2	4	A	4507.299	0.4
7	3	5	←	6	2	5	E*	4511.206	-31.4
7	3	4	←	6	2	4	E*	4514.431	-4.2
7	3	4	←	6	2	5	A	4518.693	4.6
18	3	15	←	17	3	14	E	4560.634	3.2
18	3	15	←	17	3	14	A	4561.263	13.7
18	1	17	←	17	1	16	E	4578.302	3.3
18	1	17	←	17	1	16	A	4578.672	4.0
18	2	16	←	17	2	15	E	4611.662	-10.0
18	2	16	←	17	2	15	A	4611.880	11.5
19	1	19	←	18	1	18	A	4647.307	4.7
19	1	19	←	18	1	18	E	4649.352	-7.8
19	0	19	←	18	0	18	E	4661.127	0.2
19	0	19	←	18	0	18	A	4663.200	2.3
8	3	5	←	7	2	5	E*	4761.659	17.3
8	3	6	←	7	2	6	E*	4764.890	-2.3
8	3	5	←	7	2	6	A	4773.719	2.0
19	3	16	←	18	3	15	E	4818.955	3.6
19	3	16	←	18	3	15	A	4819.412	-9.4
19	1	18	←	18	1	17	E	4825.553	-4.8
19	1	18	←	18	1	17	A	4825.925	6.2
4	4	1	←	3	3	1	E*	4856.370	2.5
4	4	1	←	3	3	0	A	4860.027	-4.7
4	4	0	←	3	3	1	A	4860.027	-6.2
19	2	17	←	18	2	16	E	4870.410	4.1
19	2	17	←	18	2	16	A	4870.633	-19.9
20	1	20	←	19	1	19	A	4889.432	-1.9
20	1	20	←	19	1	19	E	4891.926	-4.0
20	0	20	←	19	0	19	E	4900.468	-5.5
20	0	20	←	19	0	19	A	4902.979	-2.3
9	3	7	←	8	2	7	E*	5020.375	3.0
9	3	6	←	8	2	7	A	5030.369	15.3
20	4	17	←	19	4	16	A	5047.512	4.5
20	4	17	←	19	4	16	E	5048.410	-0.9
20	4	16	←	19	4	15	E	5048.972	-0.6
20	4	16	←	19	4	15	A	5050.151	11.3
20	1	19	←	19	1	18	E	5071.565	-8.0
20	1	19	←	19	1	18	A	5071.929	2.5
20	3	17	←	19	3	16	E	5078.080	0.5
20	3	17	←	19	3	16	A	5078.454	6.0
5	4	2	←	4	3	1	A	5111.764	3.9
5	4	1	←	4	3	2	A	5111.764	-6.5

5	4	1	←	4	3	1	E*	5115.117	-8.7
20	2	18	←	19	2	17	E	5128.479	-15.3
20	2	18	←	19	2	17	A	5128.804	8.6
21	1	21	←	20	1	20	A	5131.381	-3.9
21	1	21	←	20	1	20	E	5134.270	-8.8
21	0	21	←	20	0	20	E	5139.921	-1.3
21	0	21	←	20	0	20	A	5142.826	-0.2
21	4	18	←	20	4	17	A	5301.023	-2.9
21	4	18	←	20	4	17	E	5302.131	-11.5
21	4	17	←	20	4	16	E	5303.307	-4.6
21	4	17	←	20	4	16	A	5304.714	5.1
21	1	20	←	20	1	19	E	5316.328	-7.6
21	1	20	←	20	1	19	A	5316.693	4.5
21	3	18	←	20	3	17	E	5337.988	-20.4
21	3	18	←	20	3	17	A	5338.320	12.2
6	4	3	←	5	3	3	E*	5359.769	-5.7
6	4	3	←	5	3	2	A	5363.440	4.6
6	4	3	←	5	3	2	A	5363.440	4.6
22	1	22	←	21	1	21	A	5373.164	-12.1
22	1	22	←	21	1	21	E	5376.357	-13.7
22	0	22	←	21	0	21	E	5379.543	-12.3
22	0	22	←	21	0	21	A	5382.760	-1.4
22	4	19	←	21	4	18	A	5554.646	13.1
22	4	19	←	21	4	18	E	5555.890	-7.0
22	1	21	←	21	1	20	E	5559.831	-42.1
22	1	21	←	21	1	20	A	5560.238	-0.3
7	4	4	←	6	3	4	E*	5611.387	8.4
7	4	3	←	6	3	3	E*	5618.399	-14.6
23	0	23	←	22	0	22	E	5619.416	-7.2
23	0	23	←	22	0	22	A	5622.799	-0.3
23	1	22	←	22	1	21	E	5802.252	-4.7
23	1	22	←	22	1	21	A	5802.642	-10.5
23	4	20	←	22	4	19	A	5808.319	9.2
23	4	20	←	22	4	19	E	5809.581	-18.8
23	4	19	←	22	4	18	E	5813.541	-10.5
23	4	19	←	22	4	18	A	5815.154	-0.5
24	1	24	←	23	1	23	A	5856.346	-6.3
23	3	20	←	22	3	19	A	5860.238	-12.7
8	4	5	←	7	3	4	A	5866.401	-2.8
8	4	4	←	7	3	5	A	5866.710	-7.0
8	4	4	←	7	3	4	E*	5869.897	1.9
24	1	23	←	23	1	22	E	6043.573	-22.4
24	1	23	←	23	1	22	A	6044.045	0.4
24	4	21	←	23	4	20	A	6062.032	-1.5
24	4	21	←	23	4	20	E	6063.191	-24.6
24	4	20	←	23	4	19	E	6069.617	-19.6

24	4	20	←	23	4	19	A	6071.142	-3.7
25	1	25	←	24	1	24	A	6097.762	-9.3
25	0	25	←	24	0	24	E	6099.833	-13.7
25	1	25	←	24	1	24	E	6101.095	-10.4
25	0	25	←	24	0	24	A	6103.181	-11.2
9	4	6	←	8	3	6	E*	6114.185	19.7
9	4	6	←	8	3	5	A	6117.522	-6.8
9	4	5	←	8	3	6	A	6118.196	-22.0
9	4	5	←	8	3	5	E*	6121.213	17.5
24	3	21	←	23	3	20	E	6121.838	-28.0
24	3	21	←	23	3	20	A	6122.087	0.9

Bibliography

(1) Brown, G. G.; Dian, B. C.; Douglass, K. O.; Geyer, S. M.; Shipman, S. T.; Pate, B. H. A Broadband Fourier Transform Microwave Spectrometer Based on Chirped Pulse Excitation. *Rev. Sci. Instrum.* **2008**, *79* (5), 053103. https://doi.org/10.1063/1.2919120.

(2) Pérez, C.; Lobsiger, S.; Seifert, N. A.; Zaleski, D. P.; Temelso, B.; Shields, G. C.; Kisiel, Z.; Pate, B. H. Broadband Fourier Transform Rotational Spectroscopy for Structure Determination: The Water Heptamer. *Chem. Phys. Lett.* **2013**, *571*, 1–15. https://doi.org/10.1016/j.cplett.2013.04.014.

(3) Halgren, T. A. Merck Molecular Force Field. I. Basis, Form, Scope, Parameterization, and Performance of MMFF94. *J. Comput. Chem.* **1996**, *17* (5–6), 490–519. https://doi.org/10.1002/(SICI)1096-987X(199604)17:5/6<490::AID-JCC1>3.0.CO;2-P.

(4) Halgren, T. A. MMFF VI. MMFF94s Option for Energy Minimization Studies. *J. Comput. Chem.* **1999**, *20* (7), 720–729. https://doi.org/10.1002/(SICI)1096-987X(199905)20:7<720::AID-JCC7>3.0.CO;2-X.

(5) Kolossvary, I.; Keseru, G. M. Hessian-Free Low-Mode Conformational Search for Large-Scale Protein Loop Optimization: Application to c-Jun N-Terminal Kinase JNK3. *J. Comput. Chem.* **2001**, *22* (1), 21–30. https://doi.org/10.1002/1096-987X(20010115)22:1<21::AID-JCC3>3.0.CO;2-I.

(6) Maestro, Schrödinger, 2017.

(7) Becke, A. D. Density-functional Thermochemistry. I. The Effect of the Exchange-only Gradient Correction. *J. Chem. Phys.* **1992**, *96* (3), 2155–2160. https://doi.org/10.1063/1.462066.

(8) Becke, A. D. Density-functional Thermochemistry. II. The Effect of the Perdew–Wang Generalized-gradient Correlation Correction. *J. Chem. Phys.* **1992**, *97* (12), 9173–9177. https://doi.org/10.1063/1.463343.

(9) Becke, A. D. Density-functional Thermochemistry. III. The Role of Exact Exchange. *J. Chem. Phys.* **1993**, *98* (7), 5648–5652. https://doi.org/10.1063/1.464913.

(10) Grimme, S.; Antony, J.; Ehrlich, S.; Krieg, H. A Consistent and Accurate *Ab Initio* Parametrization of Density Functional Dispersion Correction (DFT-D) for the 94 Elements H-Pu. *J. Chem. Phys.* **2010**, *132* (15), 154104. https://doi.org/10.1063/1.3382344.

(11) Grimme, S.; Ehrlich, S.; Goerigk, L. Effect of the Damping Function in Dispersion Corrected Density Functional Theory. *J. Comput. Chem.* **2011**, *32* (7), 1456–1465. https://doi.org/10.1002/jcc.21759.

(12) Weigend, F.; Ahlrichs, R. Balanced Basis Sets of Split Valence, Triple Zeta Valence and Quadruple Zeta Valence Quality for H to Rn: Design and Assessment of Accuracy. *Phys. Chem. Chem. Phys.* **2005**, *7* (18), 3297. https://doi.org/10.1039/b508541a.

(13) Schäfer, A.; Huber, C.; Ahlrichs, R. Fully Optimized Contracted Gaussian Basis Sets of Triple Zeta Valence Quality for Atoms Li to Kr. *J. Chem. Phys.* **1994**, *100* (8), 5829–5835. https://doi.org/10.1063/1.467146.

(14) Frisch, M. J.; Trucks, G. W.; Schlegel, H. B.; Scuseria, G. E.; Robb, M. A.; Cheeseman, J. R.; Scalmani, G.; Barone, V.; Petersson, G. A.; Nakatsuji, H.; Li, X.; Caricato, M.; Marenich, A. V.; Bloino, J.; Janesko, B. G.; Gomperts, R.; Mennucci, B.; Hratchian, H. P.; Ortiz, J. V.; Izmaylov, A. F.; Sonnenberg, J. L.; Williams; Ding, F.; Lipparini, F.; Egidi, F.; Goings, J.; Peng, B.; Petrone, A.; Henderson, T.; Ranasinghe, D.; Zakrzewski, V. G.; Gao, J.; Rega, N.; Zheng, G.; Liang, W.; Hada, M.; Ehara, M.; Toyota, K.; Fukuda, R.; Hasegawa, J.; Ishida, M.; Nakajima, T.; Honda, Y.; Kitao, O.; Nakai, H.; Vreven, T.; Throssell, K.; Montgomery Jr., J. A.; Peralta, J. E.; Ogliaro, F.; Bearpark, M. J.; Heyd, J. J.; Brothers, E. N.; Kudin, K. N.; Staroverov, V. N.; Keith, T. A.; Kobayashi, R.; Normand, J.; Raghavachari, K.; Rendell,

A. P.; Burant, J. C.; Iyengar, S. S.; Tomasi, J.; Cossi, M.; Millam, J. M.; Klene, M.; Adamo, C.; Cammi, R.; Ochterski, J. W.; Martin, R. L.; Morokuma, K.; Farkas, O.; Foresman, J. B.; Fox, D. J. Gaussian 16 Rev. C.01, 2016.
(15) Riplinger, C.; Neese, F. An Efficient and near Linear Scaling Pair Natural Orbital Based Local Coupled Cluster Method. *J. Chem. Phys.* **2013**, *138* (3), 034106. https://doi.org/10.1063/1.4773581.
(16) Neese, F.; Wennmohs, F.; Hansen, A.; Becker, U. Efficient, Approximate and Parallel Hartree–Fock and Hybrid DFT Calculations. A ‘Chain-of-Spheres’ Algorithm for the Hartree–Fock Exchange. *Chem. Phys.* **2009**, *356* (1), 98–109. https://doi.org/10.1016/j.chemphys.2008.10.036.
(17) Neese, F.; Wennmohs, F.; Becker, U.; Riplinger, C. The ORCA Quantum Chemistry Program Package. *J. Chem. Phys.* **2020**, *152* (22), 224108. https://doi.org/10.1063/5.0004608.
(18) Pettersen, E. F.; Goddard, T. D.; Huang, C. C.; Couch, G. S.; Greenblatt, D. M.; Meng, E. C.; Ferrin, T. E. UCSF Chimera—A Visualization System for Exploratory Research and Analysis. **2004**, *25* (13), 1605–1612. https://doi.org/10.1002/jcc.20084.
(19) Hansen, N.; Mäder, H.; Bruhn, T. A Molecular Beam Fourier Transform Microwave Study of *0* -Tolunitrile: ^{14} N Nuclear Quadrupole Coupling and Methyl Internal Rotation Effects. *Mol. Phys.* **1999**, *97* (4), 587–595. https://doi.org/10.1080/00268979909482857.
(20) Pallan, P. S.; Nagy, L. D.; Lei, L.; Gonzalez, E.; Kramlinger, V. M.; Azumaya, C. M.; Wawrzak, Z.; Waterman, M. R.; Guengerich, F. P.; Egli, M. Structural and Kinetic Basis of Steroid 17α,20-Lyase Activity in Teleost Fish Cytochrome P450 17A1 and Its Absence in Cytochrome P450 17A2. *J. Biol. Chem.* **2015**, *290* (6), 3248–3268. https://doi.org/10.1074/jbc.M114.627265.
(21) Fagart, J.; Huyet, J.; Pinon, G. M.; Rochel, M.; Mayer, C.; Rafestin-Oblin, M.-E. Crystal Structure of a Mutant Mineralocorticoid Receptor Responsible for Hypertension. *Nat. Struct. Mol. Biol.* **2005**, *12* (6), 554–555. https://doi.org/10.1038/nsmb939.
(22) Bruhn, J. F.; Scapin, G.; Cheng, A.; Mercado, B. Q.; Waterman, D. G.; Ganesh, T.; Dallakyan, S.; Read, B. N.; Nieusma, T.; Lucier, K. W.; Mayer, M. L.; Chiang, N. J.; Poweleit, N.; McGilvray, P. T.; Wilson, T. S.; Mashore, M.; Hennessy, C.; Thomson, S.; Wang, B.; Potter, C. S.; Carragher, B. Small Molecule Microcrystal Electron Diffraction for the Pharmaceutical Industry–Lessons Learned From Examining Over Fifty Samples. *Front. Mol. Biosci.* **2021**, *8*, 648603. https://doi.org/10.3389/fmolb.2021.648603.
